# Evaluation of a new high-dimensional miRNA profiling platform

**DOI:** 10.1186/1755-8794-2-57

**Published:** 2009-08-27

**Authors:** Julie M Cunningham, Ann L Oberg, Pedro M Borralho, Betsy T Kren, Amy J French, Liang Wang, Brian M Bot, Bruce W Morlan, Kevin AT Silverstein, Rod Staggs, Yan Zeng, Anne-Francoise Lamblin, Christopher A Hilker, Jian-Bing Fan, Clifford J Steer, Stephen N Thibodeau

**Affiliations:** 1 Department of Laboratory Medicine & Pathology, Mayo Clinic, Rochester, Minnesota, USA; 2 Department of Health Sciences Research, Division of Biomedical Statistics and Informatics, Mayo Clinic, Rochester, Minnesota, USA; 3 iMed.UL, Faculty of Pharmacy, University of Lisbon, Lisbon 1649-003, Portugal; 4 Departments of Medicine and Genetics, Cell Biology and Development, University of Minnesota Medical School, Minneapolis, Minnesota, USA; 5 Biostatistics and Informatics, Masonic Cancer Center, University of Minnesota, Minneapolis, Minnesota, USA; 6 Department of Pharmacology, University of Minnesota, Minneapolis, Minnesota, USA; 7 Illumina Inc. 9885 Towne Centre Drive, San Diego, California, 92121, USA

## Abstract

**Background:**

MicroRNAs (miRNAs) are a class of approximately 22 nucleotide long, widely expressed RNA molecules that play important regulatory roles in eukaryotes. To investigate miRNA function, it is essential that methods to quantify their expression levels be available.

**Methods:**

We evaluated a new miRNA profiling platform that utilizes Illumina's existing robust DASL chemistry as the basis for the assay. Using total RNA from five colon cancer patients and four cell lines, we evaluated the reproducibility of miRNA expression levels across replicates and with varying amounts of input RNA. The beta test version was comprised of 735 miRNA targets of Illumina's miRNA profiling application.

**Results:**

Reproducibility between sample replicates within a plate was good (Spearman's correlation 0.91 to 0.98) as was the plate-to-plate reproducibility replicates run on different days (Spearman's correlation 0.84 to 0.98). To determine whether quality data could be obtained from a broad range of input RNA, data obtained from amounts ranging from 25 ng to 800 ng were compared to those obtained at 200 ng. No effect across the range of RNA input was observed.

**Conclusion:**

These results indicate that very small amounts of starting material are sufficient to allow sensitive miRNA profiling using the Illumina miRNA high-dimensional platform. Nonlinear biases were observed between replicates, indicating the need for abundance-dependent normalization. Overall, the performance characteristics of the Illumina miRNA profiling system were excellent.

## Background

First identified nearly 15 years ago [1], microRNAs (miRNAs) are a family of short RNA molecules that predominantly inhibit gene expression at the post-transcriptional level in eukaryotes [2,3]. In the nucleus, genes encoding primary miRNAs (pri-miRNA) are much longer than the mature form. These primary transcripts are processed by a nuclease (Drosha) and the double-stranded RNA binding protein, DGCR8, to short 60–70 nucleotide stem-loop structures (pre-miRNA). After export to the cytoplasm, pre-miRNAs are processed by interaction with the endonuclease Dicer. Mature miRNA are ~22 nucleotides in length and guide the RNA-induced silencing complex (RISC, the core components of which contain Argonaute proteins) to the target sites, usually located in the 3' untranslated region of gene transcripts[4]. Binding of the RISC leads to suppression of translation and possibly degradation of target mRNAs.

The manner in which suppression of translation is mediated is poorly understood [5]. However, it is becoming evident that this class of gene regulator controls the expression of 20–30% percent of all human genes [6]. As miRNAs regulate the expression of a large number of protein-encoding genes [7-10], a wide range of biological processes are affected, such as metabolism, organogenesis, development, cell growth, cell death, and cell fate determination. Altered expression of miRNAs has also been associated with human disease, including cancer [11-18]. Indeed, some miRNAs have been coined "oncomirs", acting in the manner of oncogenes and tumor suppressors [11,19], and oncogenes such as *Myc *have incorporated miRNA regulation into their tumorigenic potential [20].

There are now more than 541 human miRNAs identified in version 10.1 of the miRBase . For the Illumina's human miRNA BeadArray, 735 miRNA were incorporated into the beta version based on miRBase version 9.1 (February 2007 release), plus an additional 265 miRNAs derived from the literature [21]. Other than the miRNA sample preparation, the chemistry utilized by the miRNA BeadArray is similar to that used in the DASL process (**D**NA **A**nnealing, **S**election and **L**igation). The assay is highly multiplexed, using the universal Sentrix Array Matrices (SAM, Illumina).

To better understand the important physiological functions of miRNAs, high-throughput, miRNA microarray techniques have been employed to determine and compare global miRNA expression in different tissues and cell types and under different conditions [22-29]. These methods typically require micrograms of input RNA and often have a limited dynamic range. The Illumina miRNA profiling system has been described previously, showing the accuracy of the platform by rt-PCR and digital sequencing [21] using RNA from four cell lines (the same cell lines were used in this report) and purchased human tissues from Ambion. In this study, we evaluated the performance characteristics of this platform utilizing total RNA from five colon cancer cases and from four well-characterized cell lines. There is no other publication to date addressing reproducibility and performance of the Illumina miRNA profiling platform. We believe it to be highly relevant to evaluate the platform's reproducibility, and this forms the primary focus of this report. More specifically, the goals of this study were to understand variability due to plate, extraction, dilution and technical replication using cell line and clinical samples.

## Methods

### RNA Extraction

This study was approved by the Institutional Review Board of the Mayo Clinic (IRB 07-004516), and all patients provided informed consent. Tumor tissue was obtained from five patients with colon cancer. The samples were initially collected and stored in RLT buffer; total RNA was extracted using Trizol^® ^LS (Invitrogen, Corp., Carlsbad, CA). For each case, frozen tumor tissue was cut on a cryostat to generate 10-micron-thick sections. The equivalent of ~2.5-square cm of tissue at 10 microns was placed into 400 μL of RLT buffer (QIAGEN, Chatsworth, CA) including 4 μL of β-mercaptoethanol. The tube was immediately flash frozen in liquid nitrogen and then stored at -80°C until utilized for RNA extraction. 1.2 ml of Trizol^® ^LS was added to each of the vials containing the tissue sections in 400 μl of RLT, and the vials were shaken vigorously to thaw the tissue. After thawing, the 1.6 ml RLT-Trizol^® ^LS solution was divided between three 1.5-ml tubes and homogenized for 10 sec/tube using a motor-driven, disposable RNase/DNase free pestle (Thermo Fisher Scientific, Inc., Chicago, IL). The three tubes were then combined into two, resulting in 0.8 ml RLT-Trizol tissue homogenate aliquots, and incubated at room temperature for 5 min. Following the addition of 160 μl of chloroform, the tubes were shaken vigorously for 15 sec, incubated for 5 min at room temperature, followed by centrifugation at 12,000 × g for 15 min at 4°C. From each tube, 400 μl of the aqueous phase was transferred to a fresh 1.5-ml tube, 400 μl of 100% isopropanol added, mixed by vortex and incubated at room temperature for 10 min. The tubes were then centrifuged at 7,500 × g for 10 min at 4°C and the supernatant removed by pipette and 1 ml of room-temperature 75% ethanol added. Following vortex mixing, the tubes were centrifuged at 7,500 × g for 10 min at 4°C. The supernatant was removed by pipette, and the RNA pellet air dried for 5 min prior to addition of 25 μl of RNase free water. Following resuspension, RNA was quantitated by UV spectrophotometry at 260 nm, aliquoted and flash frozen in liquid nitrogen and stored at -80°C until used. One μg was examined by agarose gel electrophoresis followed by ethidium bromide staining and visualized by UV light.

Total RNA from the four cell lines used in this study was provided by Illumina. These cell lines were the following: PC-3 (prostate adenocarcinoma), MCF-7 (breast adenocarcinoma), 293 (embryonic kidney), and HeLa (cervical adenocarcinoma).

### Illumina RNA processing

The chemistry utilized by the miRNA BeadArray is similar to that used in the DASL process [30], and the workflow is available at . Varying amounts of total RNA were polyadenylated and then converted to cDNA using a biotinylated oligo-dT primer with a universal PCR sequenced at its 5' end. This was followed by annealing of a miRNA-specific oligonucleotide pool (MSO) which consists of three parts: a universal PCR priming site at the 5' end, an address sequence complementary to a capture sequence on the BeadArray and a microRNA-specific sequence at the 3' end. Extension of MSO was facilitated by addition of a polymerase, but only if their 3' bases were complementary to the cognate sequence in the cDNA template. Common primers were used to amplify the cDNA templates; the primer complimentary to the BeadArray was fluorescently labelled. The single-stranded PCR product was hybridized to the Sentrix Array Matrix (SAM), where the labelled strand binds to the bead on the array containing the complementary address sequence. The SAMs were imaged using an Illumina BeadArray Reader, which measures the fluorescence intensity at each addressed bead location. Intensity files were analyzed using BeadStudio version 3.1.1. Expression levels were expressed as an average signal value.

### Study design

**Figure 1:**
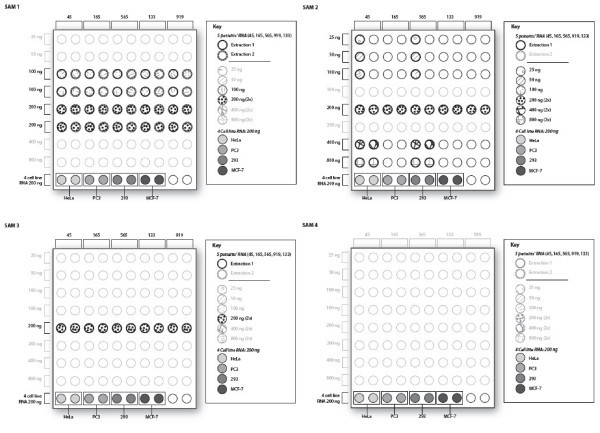
**Study design**. Sample allocation for SAMs 1–4. Five patient samples (IDs 45, 165, 565, 133 and 919) and four cell lines (HeLa, PC3, 293 and MCF-7) were utilized. Two replicates (200 ng) of each cell line RNA were assayed in SAM 1–4 (last row in each figure). In each SAM, patient IDs are shown above each column, and RNA input along each row. Blank wells contained other RNA samples not used in the present analysis. The key beside each SAM indicates samples, extraction and RNA input used. In SAM 1, two separate RNA extractions of each of the colon cancer tumors were assayed in duplicate at both 100 ng and 200 ng. In Sam 2, 25 ng–800 ng (200–800 ng each in duplicate) RNA input from extraction 1 were run for two patient IDs (45 and 565), while 200 ng was run for the other patient IDs. In SAM 3, 200 ng was run for each of the 5 colon cancer samples, each in duplicate. SAM 4 contained only the four cell line RNA.

The study used four SAMs to assess variability due to separate total RNA extractions, technical replicates of each extraction and varying total RNA input (Figure 1). Total RNA extracted from five patient samples (IDs 45, 165, 565, 919, 133) and RNA from four cell lines (HeLa, PC3, 293 and MCF-7) supplied by Illumina, was evaluated on each of 4 SAMs. SAM 1 was hybridized in week one, SAMs 2 and 3 were hybridized in week two and SAM 4 (with cell line RNA only) was hybridized in week three of the study.

Sample allocation on SAM 1 was designed to assess reproducibility between two separate total RNA extractions, between duplicate samples (technical replicates) and between two input RNA amounts. Sample allocation on SAM 2 was designed to assess technical reproducibility and the effect of six input levels of total RNA. Sample allocation on SAM 3 was designed to assess technical replication. The four cell lines, but none of the 5 patient RNA, were run on a fourth SAM. Reproducibility across all four SAMs was also assessed.

### Statistical Methods

The goals of this study were to understand variability due to SAM, extraction, dilution and technical replication. Note that normalization by definition removes systematic variation from the data. Thus, we present an initial set of analyses using un-normalized data in order to fully understand the systematic effects of interest in this study. However, since most analyses will be performed only after normalization, we also present results on normalized data. The raw miRNA expression values were exported from the BeadStudio software without background subtraction or normalization for all 735 probes. All data are presented on the log base 2 scale, where a value of 1.0 corresponds to a 2-fold change.

A common method of evaluating agreement for microarrays is via scatter plot of all expression values for sample 1 versus all expression values for sample 2. A y = x identity line is frequently overlaid on this plot along with a loess smoother and the formula for the linear regression of sample 1 on sample 2 with corresponding correlation value. Another common method of evaluating agreement is the Bland-Altman plot [31], commonly called MVA (for Minus Versus Average) or MA [32] plot in the microarray literature. For each probe, the difference between the intensities on two arrays is plotted on the vertical axis and the average of the two intensities is plotted on the horizontal axis; there is a data point for each probe on the array. While scatter plots and correlations are useful, it is generally easier to visually detect and assess magnitude of disagreement and whether the variability in disagreement is a function of abundance in an MVA plot. If two replicates yield identical results, all points will lie on the y = 0 horizontal line (indicated on the plots for reference). A loess smoother, representing a moving average, is indicated on the plots in addition to demonstrating the average bias curve as a function of average intensity. A wholesale vertical shift away from zero indicates a global mean shift between replicates. Nonlinearities in the average bias curve indicate that the bias is a function of abundance. Axis scale is kept constant throughout all MVA plots to enable comparisons of biases between plots.

It is useful to evaluate variation about the average bias curve. As noted above, the MVA plots clearly demonstrate that variability in disagreement is a function of mean abundance. Thus, the standard deviations (SD) of the differences (the vertical axis on the MVA plots) are reported separately for the probes in the bottom 50% of the abundance distribution and the top 50% of the abundance distribution. The 50^th ^percentile is generally around 9.5 to 10 on the horizontal axis. For comparison with other manuscripts, Spearman's correlations between pairs of arrays are displayed. The standard deviations and Spearman's correlations showed essentially no change from pre- to post-normalization; so only pre-normalization results are shown. Hierarchical clustering with a simple Euclidean distance metric was used as another measure of overall agreement for patient samples.

Normalizations were performed using fastlo [33], a model-based nonlinear normalization similar to cyclic loess [32]. Fastlo can be conceptualized as a loess smooth together with a simple linear model. Envision a residual MVA plot for each sample where, for a given sample, the horizontal axis corresponds to the mean over all samples (the simplest linear model); the vertical axis corresponds to the difference of this sample from the mean of all samples (i.e., residuals from this simple linear model). A loess smooth fit to this residual MVA plot corresponds to the mean bias which is subtracted out in the algorithm for each probe, allowing the bias adjustment to be a function of mean expression as recommended in Bolstad et al [34] and justified by the data presented here. Like most normalization algorithms, this algorithm assumes that most probes are not differentially expressed and that there is symmetry in the number of differentially expressed probes. Ballman et al [33] show that this implementation is equivalent to the cyclic loess implementation in [32] and that the computational time required is far less. The linear model is easily tailored to allow normalization to be performed within groups if most probes are expected to be differentially expressed and/or to accommodate labeled technologies [33,35] or other statistical experimental designs. Thus, the strengths of fastlo include an easily tailored, model-based estimation of intensity dependent biases. However, it does require knowledge of linear models in order to tailor the algorithm to specific cases. As applied here, the patient samples were normalized together since nearly all probes were expected to be similarly expressed in the five patients and between technical replicates, and no global effect due to total input RNA was observed; the cell line samples were normalized together in a separate normalization.

The detection calls generated by Illumina BeadStudio software were evaluated as part of the assessment of the effect of dilution. These are intended to be a measure of whether the signal for a probe is significantly greater than the average signal seen in the negative control probes. A cut-off of p = 0.01 was used where p < 0.01 indicates the signal was detected, as is commonly done. Venn diagrams and detection coverage plots were used to demonstrate detection overlap between dilutions and to understand differences in detection as a function of average probe abundance.

### Validation of miRNA expression results

Quantitative PCR analysis of a select number of miRNA targets was performed on the four aforementioned cell lines by Illumina [21]. Twelve probes were selected, including those with large and small differences in expression between the cell lines and those that had similar expression levels (miR-100, 125a, 125b, 135a, 146a, 150, 17-3p, 221, 26a, 31, 93 and 328). These data were produced independently from the miRNA array data and were used for validation purposes. Association of raw miRNA array expression (log2) versus the negative CT value was explored via Spearman's correlation to assess the absolute quantification capabilities of the array. All pairwise differences (log2) or fold changes (raw) between the four cell lines were also explored both within the array and qPCR data. These differences were compared in order to assess the relative quantification of the array.

## Results

Four miRNA Bead Array Plates (SAMs) were used to assess variability in patient and cell line samples due to separate total RNA extractions, technical replicates of each extraction and varying total RNA input (Figure 1).

**Figure 2:**
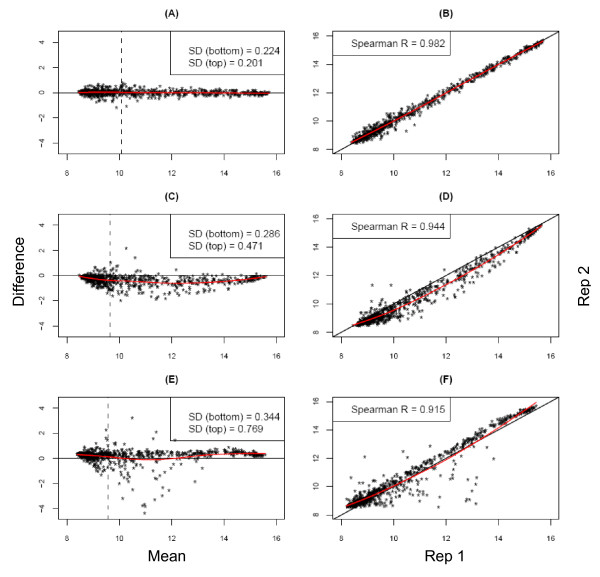
**MVA and scatter plots**. MVA plots (panels A, C, E) and the associated scatter plots (panels B, D, F) for selected technical replicates of Illumina cell line on SAM 1, chosen to demonstrate the nature of biases and variability seen in this study. All axes are on the log base 2 scale where a value of 1.0 corresponds to a 2-fold change. Panels A, C, E: For the MVA plots, the vertical axis is the difference in expression values between the two technical replicates for a given probe and the horizontal axis is the average expression between the two technical replicates. The y = 0 reference line and a loess smoother indicating the average bias as a function of abundance are indicated. The standard deviation of the differences (i.e., of the vertical axis values) is shown for the bottom and top 50% of the data with respect to average abundance as an overall indicator of variation of the plotted points. Panels B, D, F: For the scatter plots, the horizontal axis corresponds to relative expression in the first technical replicate (Rep 1), and the vertical axis the same for the second technical replicate (Rep 2). The y = x reference line and a loess smoother are indicated on the scatter plots, along with the correlation (Spearman's r) as an overall measure of agreement.

Scatter plots and the corresponding MVA plots are shown in Figure 2 for three selected technical replicates (cell lines) from SAM 1. These were chosen to provide examples of bias curves and variability observed among our test set, ranging from a low amount of scatter (overall SD 0.21) to a greater amount (overall SD 0.60). There is evidence of some disagreement between technical replicates in both the scatter and MVA plots since the points do not all fall on the respective identity lines. While the average bias smoother lies on the respective identity lines in panels A/B indicating no evidence of average bias, panels C/D and E/F show evidence of nonlinear bias with the curvature of the smoother away from the respective identity lines. Based on these plots, it is evident that the average bias can be greater than 1.0 on the log base 2 scale, corresponding to an average bias greater than 2-fold on the raw scale. The maximum disagreement is less than 2-fold in panels A/B, but is greater than 16-fold in panels E/F. The clusters of points at the far left and right ends of the MVA plot and likewise, the bottom left and top right corners of the scatter plots, show a sideways "V" pattern indicating floor or ceiling effects on the assay itself. The MVA plots show clearly that the variability around agreement is highest in the mid-expression levels, above the floor and below the ceiling, and provide a feel for the amount of scatter in the points that correspond to the respective standard deviations.

### Reproducibility between technical replicates

**Figure 3:**
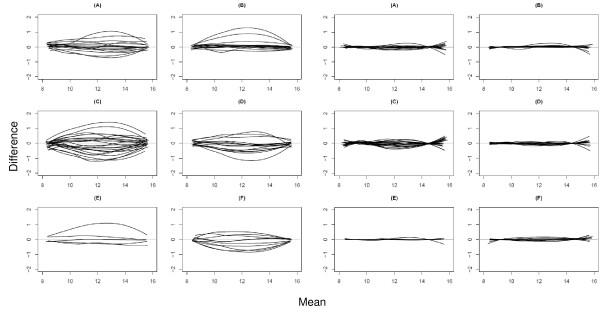
**Average bias curves**. Average bias curves (i.e. the loess smoothers) from MVA plots examining reproducibility in light of various systematic factors. The left set of six are pre-normalization; the right set of six are post-normalization. (A) Within plate technical replicates for the four cell lines on all four SAMs (n = 16 curves). (B) Within plate patient technical replicates for 200 ng of extraction 1 on first three SAMs (n = 15 curves). (C) Cell line technical replicates between SAMs (n = 24 curves). (D) Patient sample technical replicates between SAMs for 200 ng of extraction 1 (n = 15 curves). (E) Patient sample between extraction technical replicate for 200 ng (n = 5 curves). Replicate 1 was chosen arbitrarily. (F) Patient samples from extraction 1 comparing 25, 100, 400, 800 ng to 200 ng for two patients (n = 10 curves). Replicate 1 for 200 ng was chosen arbitrarily.

**Figure 4:**
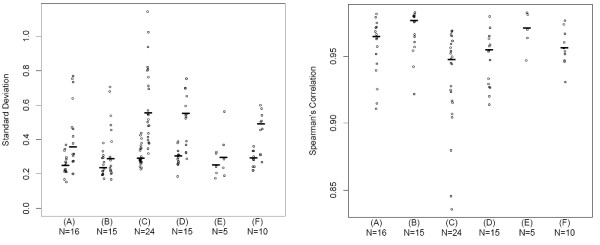
**Standard deviations and correlations**. Left box: Standard deviations (SD) summarizing variability in probe differences corresponding to the bias curves in Figure 3. The left column of points for each panel is for the low abundance probes, the right column of points is for the high abundance probes. A horizontal mark indicates the median SD for each column. Right box: Spearman's correlation values, r, assessing association of replicates. A horizontal line indicates the median r value for each panel. These data change little after normalization (data not shown). The descriptions for sections A – F within the boxes are the same as those described for Panels A – F in Figure 3.

Average bias curves for the within-plate cell line technical replicates on all four SAMs (n = 16 curves) and then for the 200 ng replicates of the first extraction for the five CRC samples on the first three SAMs (n = 15 curves) are displayed in panels A and B, respectively, of the left set of six plots in Figure 3. Post-normalization bias curves are shown in the same panels of the right set of six plots in Figure 3. Please note that Figures 3 and 4 have multiple panels where each panel corresponds to a different type of replication. The mapping of panels and labels to replication type is consistent for all of these figures for ease of comparison. As variability in disagreement is a function of mean abundance, the SD of the differences was calculated for both the bottom half and the top half of the data with respect to abundance. This corresponds to drawing a vertical line on each MVA plot at the median average abundance value. The median average abundance is generally around 9.5 to 10. The actual data points would make the plot illegible and are thus not shown. All MVA plots with data points are in the supplementary figures (see Additional files A1, A2, A3, A4, A5, and A6). Results are shown only for the 200 ng replicates and were similar for the 100 ng hybridizations. For many technical replicates the average bias is fairly linear and small indicating good reproducibility. However, some bias curves show average bias reaching approximately 1.0, corresponding to a 2-fold change on the raw scale as well as nonlinearity indicating the need for nonlinear normalization. As seen in the right-hand set of plots in Figure 3, the average bias has been removed after normalization.

The standard deviations displayed in panels A and B of the left box of Figure 4 summarize the variability of the points corresponding to the MVA plots for the technical replicates. The low-abundance probes (left columns) consistently have lower variation than the high-abundance probes. Many of the low-abundance probes may not be significantly higher than background, so this variation may be a measure of random background noise. The distributions for low- and high-abundance probes are similar for both the cell line and patient technical replicates. Spearman's correlations are shown in the right box of Figure 4 in panels A and B. The ranges of values are similar for cell line and patient data, ranging from 0.91 to 0.98. Overall, these data show good technical reproducibility within SAMs and a need for non-linear normalization.

### Reproducibility between SAMs

**Figure 5:**
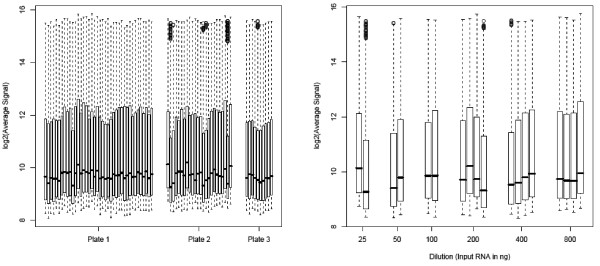
**Signal distribution by plate and input RNA**. Left panel: Pre-normalization box and whisker plots depicting distribution of probe intensities for technical replicates on SAMs 1, 2, and 3. The vertical axis is log base 2 intensity and the horizontal axis is SAM well ID. The bottom and top of the box represent the 25^th ^and 75^th ^percentiles of the probe expression values. The horizontal bar inside the box represents the median. The whiskers extend to either 1.5 times the interquartile range (75^th ^percentile minus the 25^th ^percentile) or the most extreme point, whichever is shorter. Points beyond the whiskers indicate outliers. No global shift in distribution is seen between plates. Right panel: Pre-normalization box and whisker plots depicting distribution of probe intensities at various amounts of starting material. The vertical axis corresponds to a log base 2 intensity, while the horizontal axis corresponds to the ng of starting material.

Technical replicates for cell line and human samples were then assessed between SAMs. Pre-normalization box and whisker plots are shown in the left panel of Figure 5. There is no clear global shift in expression across the different SAMs. There are a few wells on SAM 2 that have more outliers than other wells. The first replicate was arbitrarily chosen for MVA plots comparing replicates between SAMs. The bias curves comparing replicate 1 intensities on a SAM to the replicate intensities on another SAM for all possible pairs of SAMs are displayed in Figure 3 (24 curves in panel C for cell lines and 15 curves in panel D for CRC subjects). The average bias curves show greater biases within SAM technical replicate curves, extending up to 1.3 (nearly 2.5-fold change) in addition to many small biases. Within the individual MVA plots, it is evident that the agreement is better between SAMs 1 and 3 than between SAMs 1 or 3 and 2; there are a few oddly behaving probes on SAM 2 (see Additional files A3 and A4). The distribution of standard deviations of the probe differences is shifted slightly higher than for technical replicates within a SAM for the low-abundant data and shifted more dramatically for the high-abundant data (Figure 4, left box, panels C and D). Likewise, the distribution of Spearman's correlations is shifted down, indicating slightly weaker agreement between SAMs than within SAMs (Figure 4, right box, panels C and D). These values ranged from 0.84 to 0.98. For most, the correlation remains above 0.9. After normalization the biases are mostly removed (Figure 3, panels C and D of the right set of plots). Overall, while the reproducibility between SAMs is not as good as that within SAMs, it is acceptable.

### Reproducibility between extractions

Two separate total RNA extractions were carried out on five CRC samples. In order to compare between extractions, technical replicate 1 within an extraction, was chosen without loss of generality for presentation here. Bias curves comparing the intensities for extraction 1 versus the intensities of extraction 2 for each patient sample are displayed in panel E of Figure 3 (pre-normalization, left set; post-normalization, right set). These curves indicate relatively small average biases between extractions for most patients. Standard deviations for the differences between extractions (Figure 4, left box, panel E) in the low-abundant probes are similar to those within an extraction (panel B); values for the high-abundant data are remarkably similar to those within an extraction with similar median values. Likewise, the Spearman's correlations (Figure 4, right box, panel E) are high, ranging from 0.95 to 0.98. Post-normalization, the biases are again nearly completely removed. Clusters on pre-normalization data are not grouped by patient, extraction or dilution (100 or 200 ng) (see Additional file 7). However, post-normalization data are grouped by patient and not dilution or extraction, indicating similar expression profiles for each patient regardless of extraction or dilution (see Additional file 8). Between-extraction reproducibility is comparable to within-SAM reproducibility in these limited data.

### Effect of varying input of RNA

As it is likely that many clinical samples will have limited amounts of tissue, and hence RNA, available for assessing miRNA expression profiling, we examined whether reliable results could be attained when input was as low as 25 ng. Two of the CRC samples (IDs 565 and 45) were run with one replicate of 25, 50 and 100 ng and two technical replicates of 200, 400 and 800 ng of total RNA on SAM 2. There is little difference between dilutions as evidenced by box and whisker plots; there is no global shift in intensity across input amount as would be expected if there was a dilution effect (Figure 5, right panel). Before normalization, the average bias curves of each dilution versus the 200 ng dilution in panel F of Figure 3 (left set) showed no evidence of a shift in abundance, since the curves are mostly centered about zero rather than the corresponding expected fold changes. Most curves show little nonlinearity. The distribution of standard deviations for both the low- and high-abundant data (Figure 4, left box, panel F) are shifted higher than for other systematic sources of variation indicating greater variability in disagreement. Likewise, the Spearman's correlations are shifted lower (Figure 4, right box, panel F) with a median value of 0.95 suggesting weaker agreement. After normalization, the nonlinear biases are mostly removed (Figure 3, panel F of right set). Clusters on both pre- and post-normalization data are not grouped by either patient or dilution (100 or 200 ng) (see Additional files 9 and 10). These data indicate that microRNA profiling using this chemistry can be achieved with as little as 25 ng of total RNA.

It is of interest to compare the detection rates between dilutions. The Illumina software defines a detection p-value for a probe by comparing the probe intensity to the distribution of the background probes. We set a cut-off of p = 0.01 to determine whether a probe was "significantly detected". For the two patients, 53% (386/735) and 51% (377/735) of the probes were detected in both 200 ng technical replicates, while 12% (88/735) and 5% (39/735) were detected in only one of the replicates. Venn diagrams show the overlap in detection with replicate 1 of 200 ng with all other dilutions (see Additional file A11). Overall, no systematic differences were noted in the number detected or not detected across the dilutions. Additionally, of the 461 probes detected in replicate 1 of the 200 ng dilution for one patient, 75%, 10%, 4%, 7%, 3%, and 2% were also detected in 5, 4, 3, 2, 1 or 0 of the other 5 dilutions for the one patient. Thus, 89% of the probes detected at 200 ng were detected in three or more of the other dilutions. Of the 274 probes not detected at 200 ng, 0%, 1%, 3%, 6%, 14% and 75% were detected in 5, 4, 3, 2, 1 or 0 other dilutions. Thus, of the probes not detected at 200 ng, only 4% were detected in three or more dilutions. These percentages are similar for the second patient.

**Figure 6:**
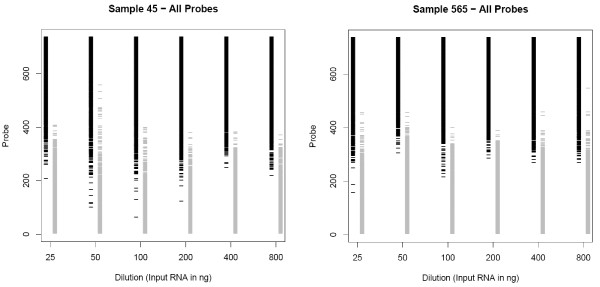
**Probe signal detection**. Detection coverage plots for both patients showing overlap in probe detection between dilutions. The two panels correspond to the two patients used for the dilution assessment. The horizontal axis corresponds to dilution, or amount of input RNA. For the vertical axis, the average intensity across the six dilutions was calculated for each patient separately. These average values were then ranked in abundance from 1 (lowest average abundance) to 735 (highest average abundance). The vertical axis corresponds to this ranking. The left column for each dilution corresponds to probes detected; and a black horizontal line is drawn only if a probe *is *detected in that dilution; white space if a probe is not detected. Due to the density of the probes in the column, a second column is included for each dilution indicating negative detection calls; a gray horizontal line is drawn if the probe is *not *detected; white space if a probe is detected. Thus, a probe detected (or not detected) in all dilutions would have a black (or gray) line in all dilutions.

A visual depiction of the variation in detection across dilutions is provided in Figure 6. In this figure, the probes are arranged vertically according to their relative average abundance across dilutions. The left column for each dilution corresponds to detectable probes; a black horizontal line is drawn if a probe *is *detected in that dilution; if it is not detected, no line is drawn (white space). Due to the density of the probes in the column, a second column is included for each dilution indicating negative detection calls; a gray horizontal line is drawn if the probe was *not *detected; and a white space if a probe was detected. Thus, a probe detected (or not detected) in all dilutions would have a black (or gray) line in all dilutions. Thus, it is possible to visually track the presence (black) or absence (gray) of individual probes across each dilution. The solid black columns and corresponding lack of grey lines for probes with high-abundance rank indicates that probes with the highest abundance levels are consistently detected in all dilutions. Likewise, the lack of black lines at the low-abundance probes and the solidness of the grey columns indicate the low-ranking abundance probes are consistently not detected in all dilutions. Thus, the likelihood of a positive detection is clearly related to average abundance. If likelihood of probe detection was related to dilution, one would expect either the depth of the black columns (and correspondingly the height of the grey columns) to follow an increasing trend with increasing dilution or a decrease in number of grey lines overall with increasing dilution. However, the point at which the black columns begin to give way to white space is remarkably consistent across dilutions for both patients. Evaluating the data from these two patients, there is no clear trend in detection over dilution. Thus, as noted above, as little as 25 ng of total RNA may be used as input for the Illumina microRNA expression profiling.

### Validation of miRNA data using qPCR

**Figure 7:**
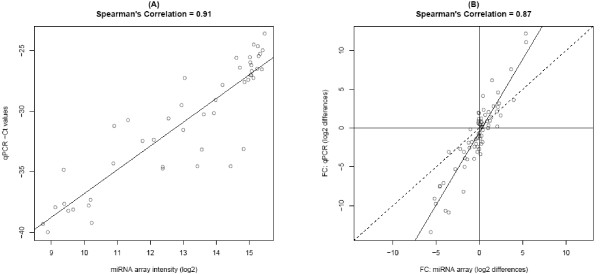
**Correlation of array and qPCR results**. (A) A scatterplot illustrating the association of raw miRNA array expression (log2) versus the negative CT value (Spearman's correlation 0.91) is used to assess the quantification capabilities of the array in a single sample. (B) All pairwise differences (log2) between the four cell lines were computed both within the array and qPCR data and plotted to assess the quantification capabilities of the array for relative differences between samples (Spearman's correlation 0.87). Included in the scatterplot are both the dotted y = x line as well as the solid regression fit line (y = -0.56 + 1.88x).

For validation of miRNA expression, qPCR data was explored for 12 miRNAs that were chosen based on varying degrees of differential expression between the four cell lines as observed in a separate miRNA array experiment. Absolute expression from the miRNA array appeared to correlate well with qPCR negative CT values (Figure 7A: Spearman's correlation of 0.91). All pairwise differences between cell lines within each miRNA target were also explored to determine the relative expression patterns of the array. Results also correlated well in this comparison (Figure 7B: Spearman's correlation 0.87), although fold change differences in miRNA array expression were consistently lower than those of qPCR as is illustrated by the slope of the regression line.

## Discussion

This report is aimed at understanding the performance characteristics of a newly developed, highly sensitive, high-dimensional miRNA expression platform, the Illumina human miRNA BeadArray. In particular, we wished to rigorously assess reproducibility of the assay and present raw, un-normalized data to better understand the performance of the assay. Overall, the Illumina miRNA array system performed very well. With respect to reproducibility (both within a plate and between plates), this assay was found to be similar to other high-dimensional gene expression platforms. The recommended input of RNA was 200 ng. Using this as the reference, however, we found no evidence for a dilution effect, with comparable results obtained from 25 ng to 800 ng RNA. Therefore, miRNA from small tissue samples can be reliably assayed. Additionally, this platform can test up to 96 samples at a time, providing high throughput capability. A description of the platform has been previously published [21] and included validation of 12 individual miRNA by qPCR, and on a more global scale, miRNA expression was validated using digital gene expression.

The experimental approach was to look at variability between extractions, between technical replicates (both within and between plates) and between varying inputs of RNA using RNA from five colon cancer specimens and four cell lines. The agreement between extractions appears to be similar to that between technical replicates within an extraction and was similar for all the samples tested. As one would expect, the between-plate agreement was slightly less than within-plate agreement, although most correlation values were >0.90. There were several outliers noted in one SAM. Processed at the same time as another SAM, these may be related to sample handling or perhaps to a few underperforming probes.

We present both scatter plots and MVA plots to evaluate the data; the latter are a useful tool for assessing biases in high-dimensional data. Just as with high-dimensional gene expression microarray platforms [32,36,34,38,37], the bias curves observed here demonstrate that bias is not constant across all abundance levels for high-dimensional microRNA data indicating a need for nonlinear normalization. There is debate in current literature as to whether normalization algorithms used for high density mRNA microarrays are applicable to data from microRNA microarrays [39-43]. Most commonly used mRNA normalization algorithms assume that only a small portion of probes are differentially expressed, that the distribution of differentially expressed probes is approximately symmetric about identity. In addition, there must be sufficient probes for estimation of bias without over-fitting. These assumptions must be evaluated specific to the experiment at hand. The data we present here suggest that there are sufficient numbers of probes expressed on this microRNA platform for estimation of biases without over-fitting using standard mRNA normalization algorithms in this experiment. In addition, there is no differential expression expected in the replicates analyzed herein. Thus, the assumptions hold for these data and nonlinear normalizations such as quantile [34,44] and cyclic loess [32,33] should be directly applicable to this experiment. Indeed, the fastlo normalization method utilized in this report removed the nonlinearity seen in the bias curves.

Finally, we selected 12 miRNA targets to further assess the accuracy of the Illumina array utilizing a second method to quantitate miRNA levels (qPCR). Using RNA from the four cells lines used in this study, the correlation between miRNA expression levels derived from the Illumina platform compared to that from the qPCR analysis was excellent for the 12 targets. These results were essentially the same as those previously reported [21].

Overall, the Illumina array appears to be quite specific for the mature form of the miRNA. Chen et al. hypothesize that the cDNA synthesis may be more complete for the short mature miRNAs than for the pre-miRNA templates. Also, the stem-loop structure of pre-miRNAs could hinder the cDNA synthesis and annealing of the oligonucleotides, resulting in relatively selective detection of expression of the mature miRNAs [21].

In summary, we found this high-dimensional miRNA profiling platform to be highly sensitive, providing reproducible data over a wide range of RNA input amounts. The variability between extraction, replicates, and SAMs was found to be acceptable. After comparison with independently produced qPCR data, validation of the absolute and relative quantification of the technology seemed adequate as well.

## Conclusion

Illlumina's miRNA profiling application provides an excellent tool for determining miRNA expression in clinical and research samples. Small amounts of RNA may be used to generate highly reproducible data. The Illumina miRNA panel therefore, presents a robust tool for a variety of research applications, providing advantages over existing tools

## Competing interests

The authors declare that they have no competing interests.

## Authors' contributions

CJS and SNT conceived the project. CJS, SNT, AJF, JMC, ALO, BMB, BWB participated in the study design. JMC carried out the miRNA profiling, assisted by CAH, and validation data were provided by JBF. ALO was responsible for analysis of the data; assisted by BMB and BWM. AJF, LW, PMB and BTK participated in extracting RNA from the tissue samples. JMC and ALO drafted the manuscript. KATS, RS, YZ, AFB were involved in analysis of early data and in editing of the manuscript. All authors read and approved the final manuscript.

## Pre-publication history

The pre-publication history for this paper can be accessed here:



## Supplementary Material

Additional file 1 **MVA plots: within plate cell line replicates**. Pre-normalization MVA plots for within plate cell line technical replicates on all four SAMs corresponding to panel A of Figures 3 and 4. Axes are described in the manuscript. Click here for file

Additional file 2 **MVA plots: within plate patient replicates**. Pre-normalization MVA plots for within plate patient technical replicates for 200 ng of extraction 1 on the first three SAMs corresponding to panel B of Figures 3 and 4. Axes are described in the manuscript. Click here for file

Additional file 3 **MVA plots: between plate cell line replicates**. Pre-normalization MVA plots for 200 ng between SAM cell line technical replicates corresponding to panel C of Figures 3 and 4. Axes are described in the manuscript. Click here for file

Additional file 4 **MVA plots: between plate patient replicates**. Pre-normalization MVA plots for 200 ng between SAM patient technical replicates for 200 ng of extraction 1 corresponding to panel D of Figures 3 and 4. Axes are described in the manuscript. Click here for file

Additional file 5 **MVA plots: between extractions**. Pre-normalization MVA plots for 200 ng between extraction patient technical replicates for 200 ng corresponding to panel E of Figures 3 and 4. Axes are described in the manuscript. Click here for file

Additional file 6 **MVA plots: between dilutions**. Pre-normalization MVA plots for patient samples from extraction 1 comparing 25, 100, 400, 800 ng to 200 ng for two patients corresponding to panel F of Figures 3 and 4. Axes are described in the manuscript. Click here for file

Additional file 7 **Pre-normalization clustering dendrogram, plate 1**. Pre-normalization dendrogram depicting the results of clustering performed on patient samples on plate 1. Sample IDs are of the form T*dilution.ptID.extraction.replicate*. For example, T200.133.1a.1 represents the 200 ng dilution for patient 133 from extraction 1, replicate 1. Click here for file

Additional file 8 **Post-normalization clustering dendrogram, plate 1**. Post-normalization dendrogram depicting the results of clustering performed on patient samples on plate 1. Sample IDs are as described for Additional file 7. Click here for file

Additional file 9 **Pre-normalization clustering dendrogram, plate 2**. Pre-normalization dendrogram depicting the results of clustering performed on patient samples on plate 2. Sample IDs are as described for Additional file 7. Click here for file

Additional file 10 **Post-normalization clustering dendrogram, plate 2**. Post-normalization dendrogram depicting the results of clustering performed on patient samples on plate 2. Sample IDs are as described for Additional file 7. Click here for file

Additional file 11 **Venn diagrams of signal detection between replicates**. Venn diagrams showing overlap in detection calls between each dilution and the 200 ng replicate 1 for both patients with dilution replicates on SAM 2. Each box represents comparison of one dilution versus the 200 ng replicate 1 sample, where the dilution is labeled on the tops of circles within a box. The numbers inside the circles indicate the number of probes detected in the 200 dilution, both dilutions, or the comparison using the p = 0.01 cut-off to determine detection. The numbers in the bottom right of each box indicate the number of probes not detected in either dilution. For example, for patient 45 comparing 200 ng versus 25 ng, 388 probes were detected in both dilutions, 73 or 16 probes were detected in only the 200 ng or 25 ng dilution, respectively, and 258 were not detected in either dilution. Click here for file
