# Supplementary material for: Evaluation of a new high-dimensional miRNA profiling platform
Source: BMC Med Genomics. 2009 Aug 27;2:57. doi: 10.1186/1755-8794-2-57 (PMC2744682; doi:10.1186/1755-8794-2-57)
Supplement: Additional file 1 — MVA plots: within plate cell line replicates. Pre-normalization MVA plots for within plate cell line technical replicates on all four SAMs corresponding to panel A of Figures 3 and 4. Axes are described in the manuscript. [file 1755-8794-2-57-S1.pdf]

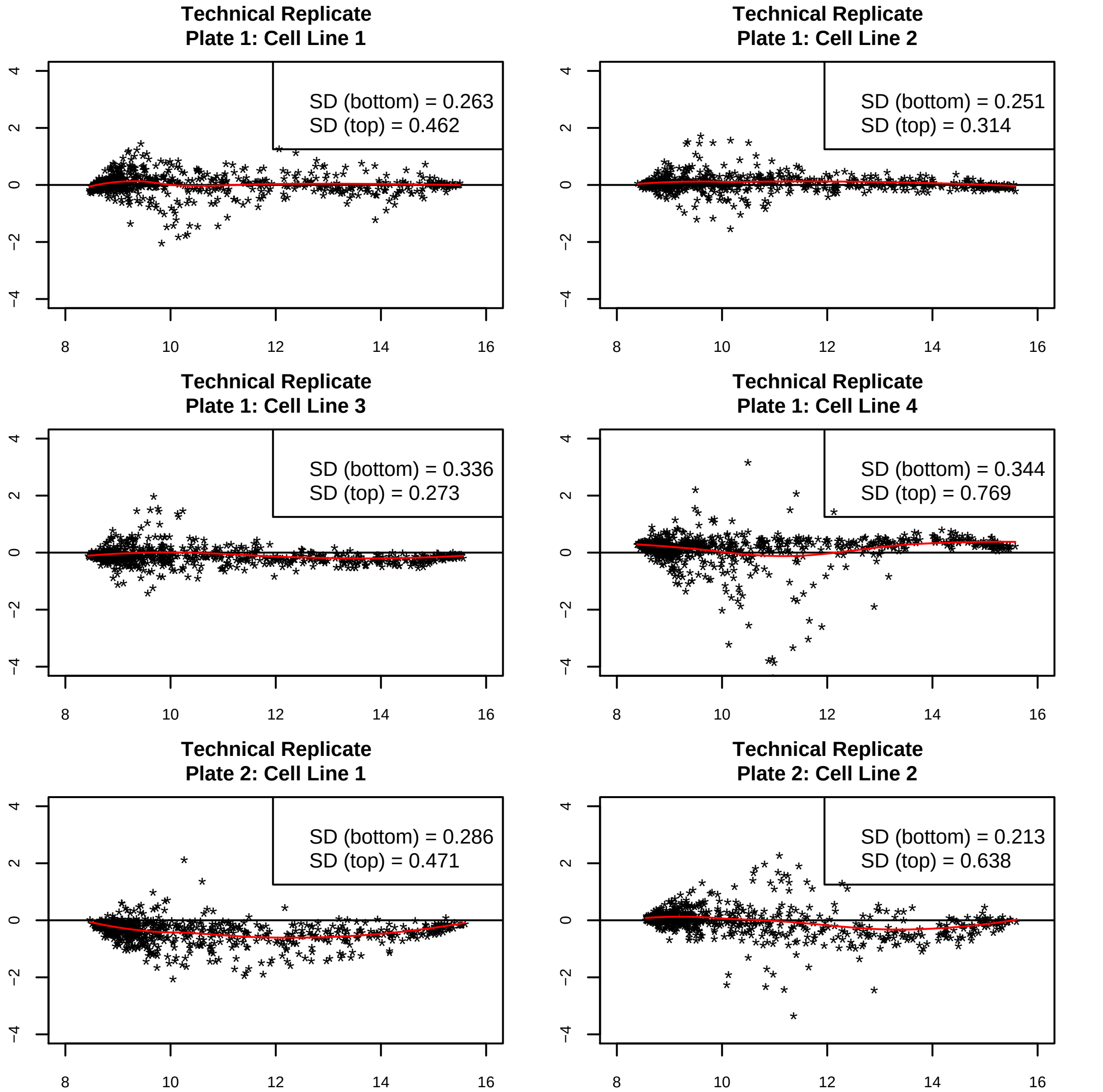
Technical Replicate
Plate 1: Cell Line 1
SD (bottom) = 0.263
SD (top) = 0.462
Technical Replicate
Plate 1: Cell Line 2
SD (bottom) = 0.251
SD (top) = 0.314
Technical Replicate
Plate 1: Cell Line 3
SD (bottom) = 0.336
SD (top) = 0.273
Technical Replicate
Plate 1: Cell Line 4
SD (bottom) = 0.344
SD (top) = 0.769
Technical Replicate
Plate 2: Cell Line 1
SD (bottom) = 0.286
SD (top) = 0.471
Technical Replicate
Plate 2: Cell Line 2
SD (bottom) = 0.213
SD (top) = 0.638
−4
−2
0
2
4
8
10
12
14
16

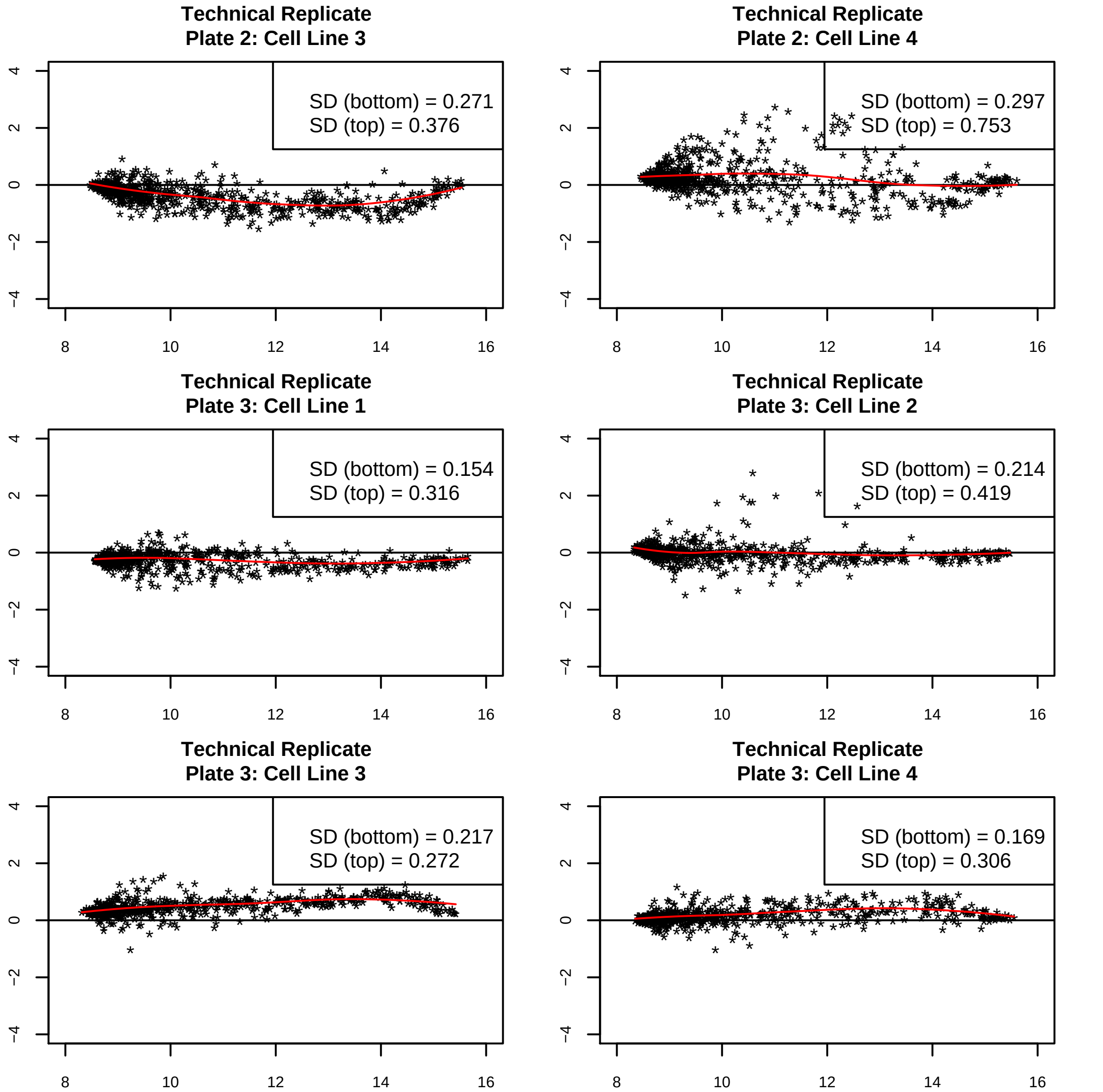
Technical Replicate
Plate 2: Cell Line 3
SD (bottom) = 0.271
SD (top) = 0.376
Technical Replicate
Plate 2: Cell Line 4
SD (bottom) = 0.297
SD (top) = 0.753
Technical Replicate
Plate 3: Cell Line 1
SD (bottom) = 0.154
SD (top) = 0.316
Technical Replicate
Plate 3: Cell Line 2
SD (bottom) = 0.214
SD (top) = 0.419
Technical Replicate
Plate 3: Cell Line 3
SD (bottom) = 0.217
SD (top) = 0.272
Technical Replicate
Plate 3: Cell Line 4
SD (bottom) = 0.169
SD (top) = 0.306

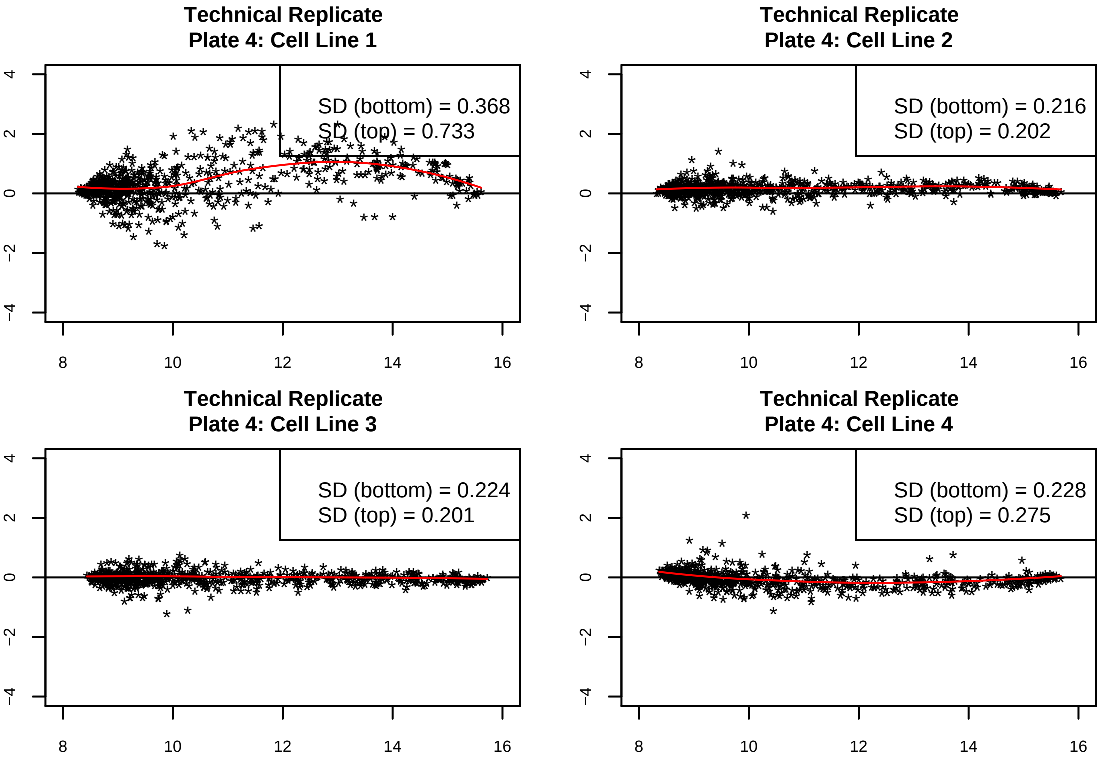
Technical Replicate
Plate 4: Cell Line 1
SD (bottom) = 0.368
SD (top) = 0.733
Technical Replicate
Plate 4: Cell Line 2
SD (bottom) = 0.216
SD (top) = 0.202
Technical Replicate
Plate 4: Cell Line 3
SD (bottom) = 0.224
SD (top) = 0.201
Technical Replicate
Plate 4: Cell Line 4
SD (bottom) = 0.228
SD (top) = 0.275
4
2
0
−2
−4
8
10
12
14
16
