# Supplementary material for: Evaluation of a new high-dimensional miRNA profiling platform
Source: BMC Med Genomics. 2009 Aug 27;2:57. doi: 10.1186/1755-8794-2-57 (PMC2744682; doi:10.1186/1755-8794-2-57)
Supplement: Additional file 2 — MVA plots: within plate patient replicates. Pre-normalization MVA plots for within plate patient technical replicates for 200 ng of extraction 1 on the first three SAMs corresponding to panel B of Figures 3 and 4. Axes are described in the manuscript. [file 1755-8794-2-57-S2.pdf]

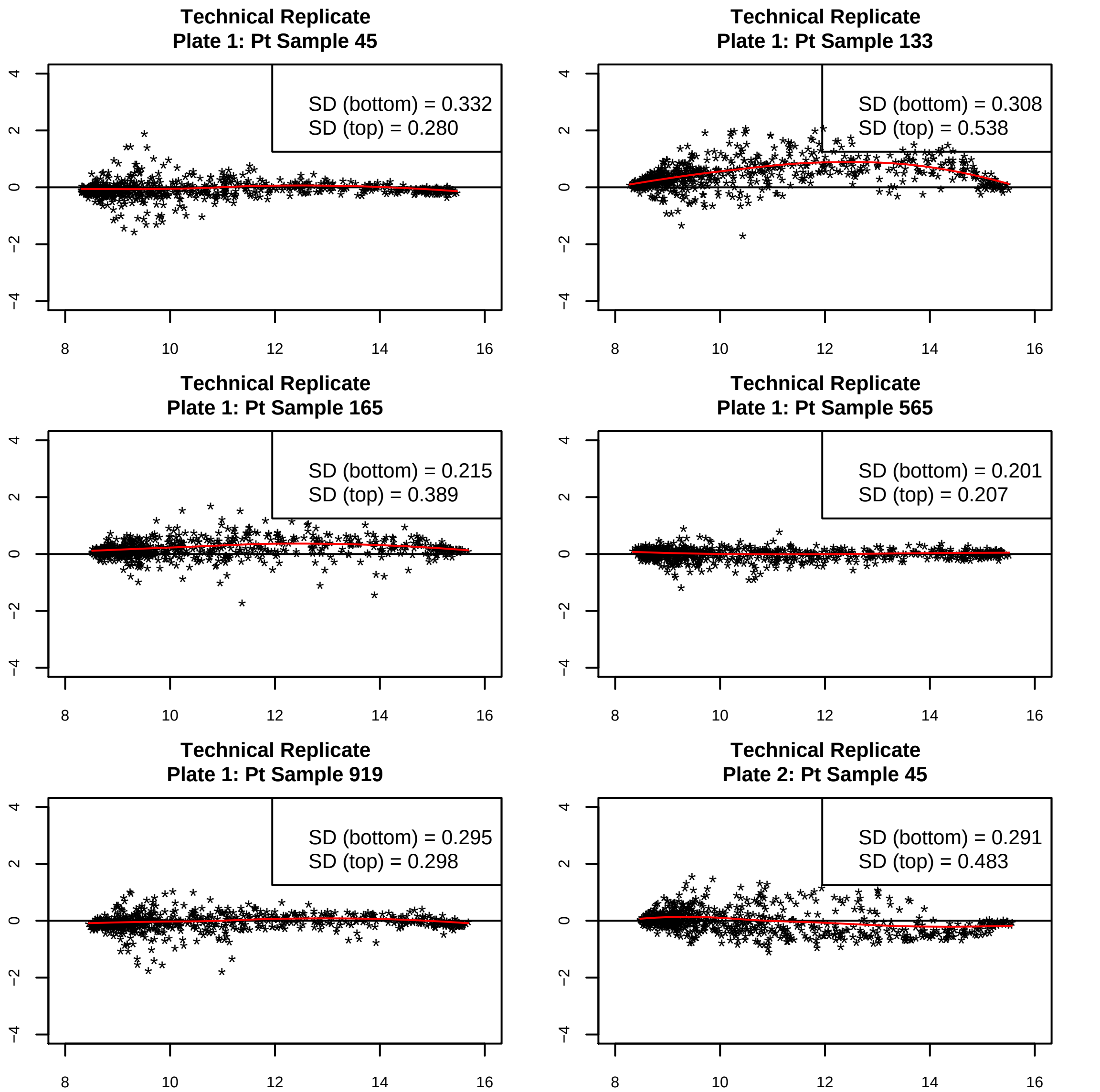

Technical Replicate
Plate 1: Pt Sample 45
SD (bottom) = 0.332
SD (top) = 0.280
Technical Replicate
Plate 1: Pt Sample 133
SD (bottom) = 0.308
SD (top) = 0.538
Technical Replicate
Plate 1: Pt Sample 165
SD (bottom) = 0.215
SD (top) = 0.389
Technical Replicate
Plate 1: Pt Sample 565
SD (bottom) = 0.201
SD (top) = 0.207
Technical Replicate
Plate 1: Pt Sample 919
SD (bottom) = 0.295
SD (top) = 0.298
Technical Replicate
Plate 2: Pt Sample 45
SD (bottom) = 0.291
SD (top) = 0.483
4
2
0
−2
−4
8
10
12
14
16

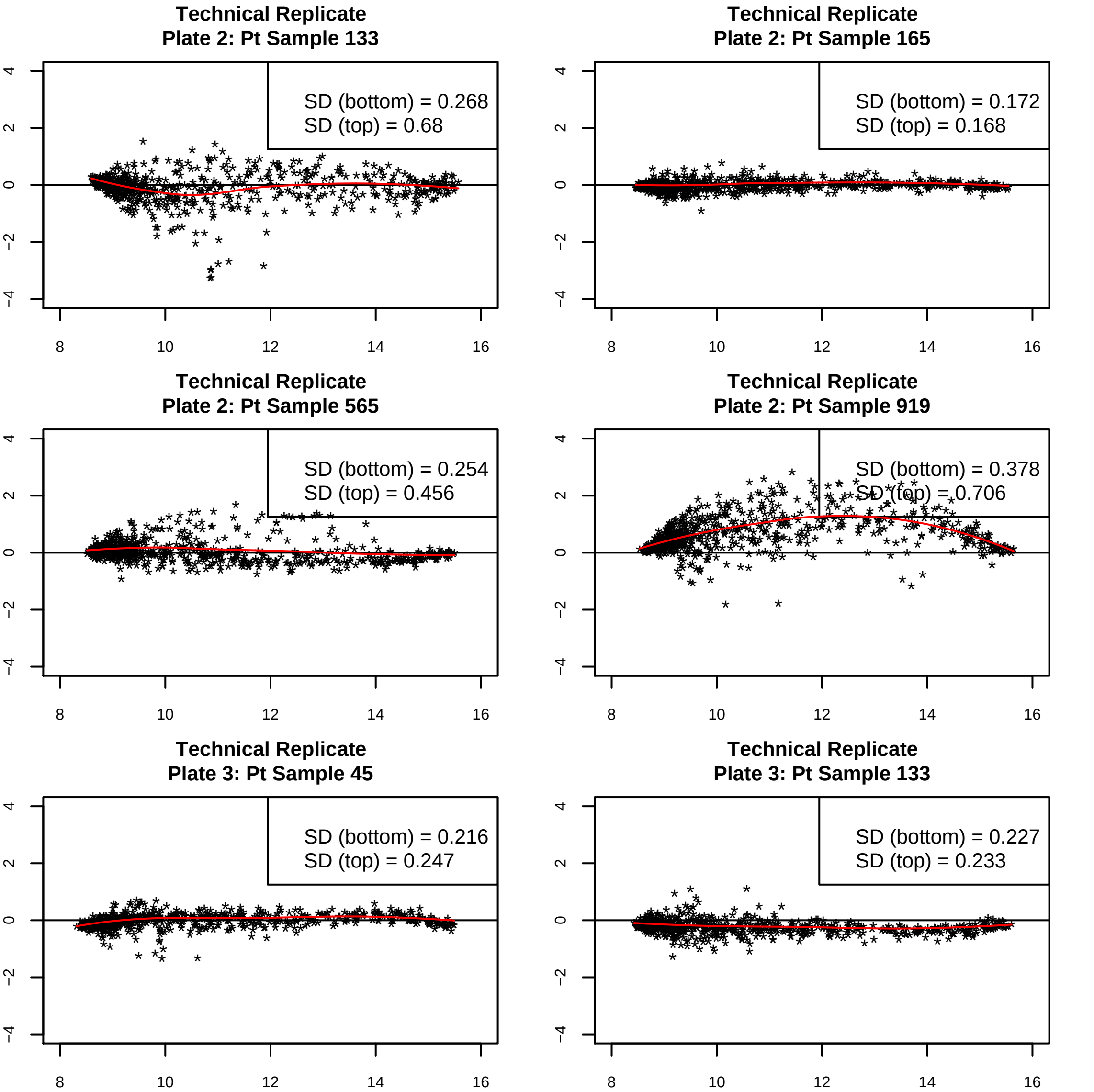
Technical Replicate
Plate 2: Pt Sample 133
SD (bottom) = 0.268
SD (top) = 0.68
Technical Replicate
Plate 2: Pt Sample 165
SD (bottom) = 0.172
SD (top) = 0.168
Technical Replicate
Plate 2: Pt Sample 565
SD (bottom) = 0.254
SD (top) = 0.456
Technical Replicate
Plate 2: Pt Sample 919
SD (bottom) = 0.378
SD (top) = 0.706
Technical Replicate
Plate 3: Pt Sample 45
SD (bottom) = 0.216
SD (top) = 0.247
Technical Replicate
Plate 3: Pt Sample 133
SD (bottom) = 0.227
SD (top) = 0.233
4
2
0
−2
−4
8
10
12
14
16

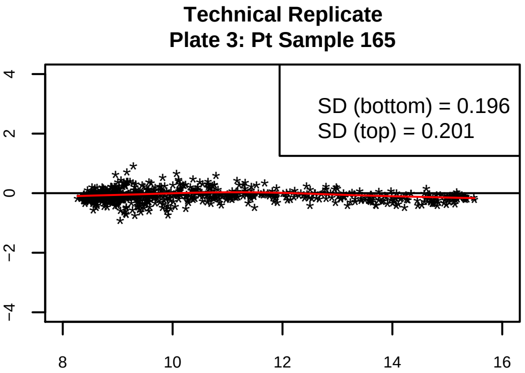
Technical Replicate
Plate 3: Pt Sample 165
SD (bottom) = 0.196
SD (top) = 0.201
4
2
0
−2
−4
8
10
12
14
16

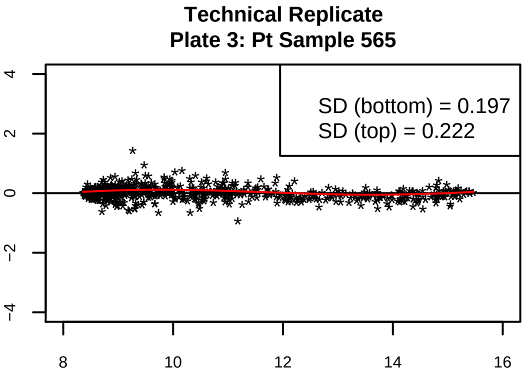
Technical Replicate
Plate 3: Pt Sample 565
SD (bottom) = 0.197
SD (top) = 0.222
4
2
0
−2
−4
8
10
12
14
16

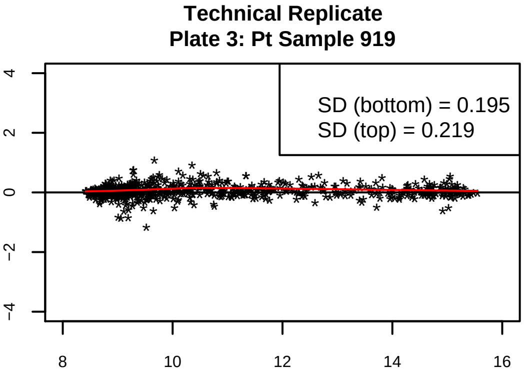
Technical Replicate
Plate 3: Pt Sample 919
SD (bottom) = 0.195
SD (top) = 0.219
4
2
0
−2
−4
8
10
12
14
16
