# Supplementary material for: Evaluation of a new high-dimensional miRNA profiling platform
Source: BMC Med Genomics. 2009 Aug 27;2:57. doi: 10.1186/1755-8794-2-57 (PMC2744682; doi:10.1186/1755-8794-2-57)
Supplement: Additional file 3 — MVA plots: between plate cell line replicates. Pre-normalization MVA plots for 200 ng between SAM cell line technical replicates corresponding to panel C of Figures 3 and 4. Axes are described in the manuscript. [file 1755-8794-2-57-S3.pdf]

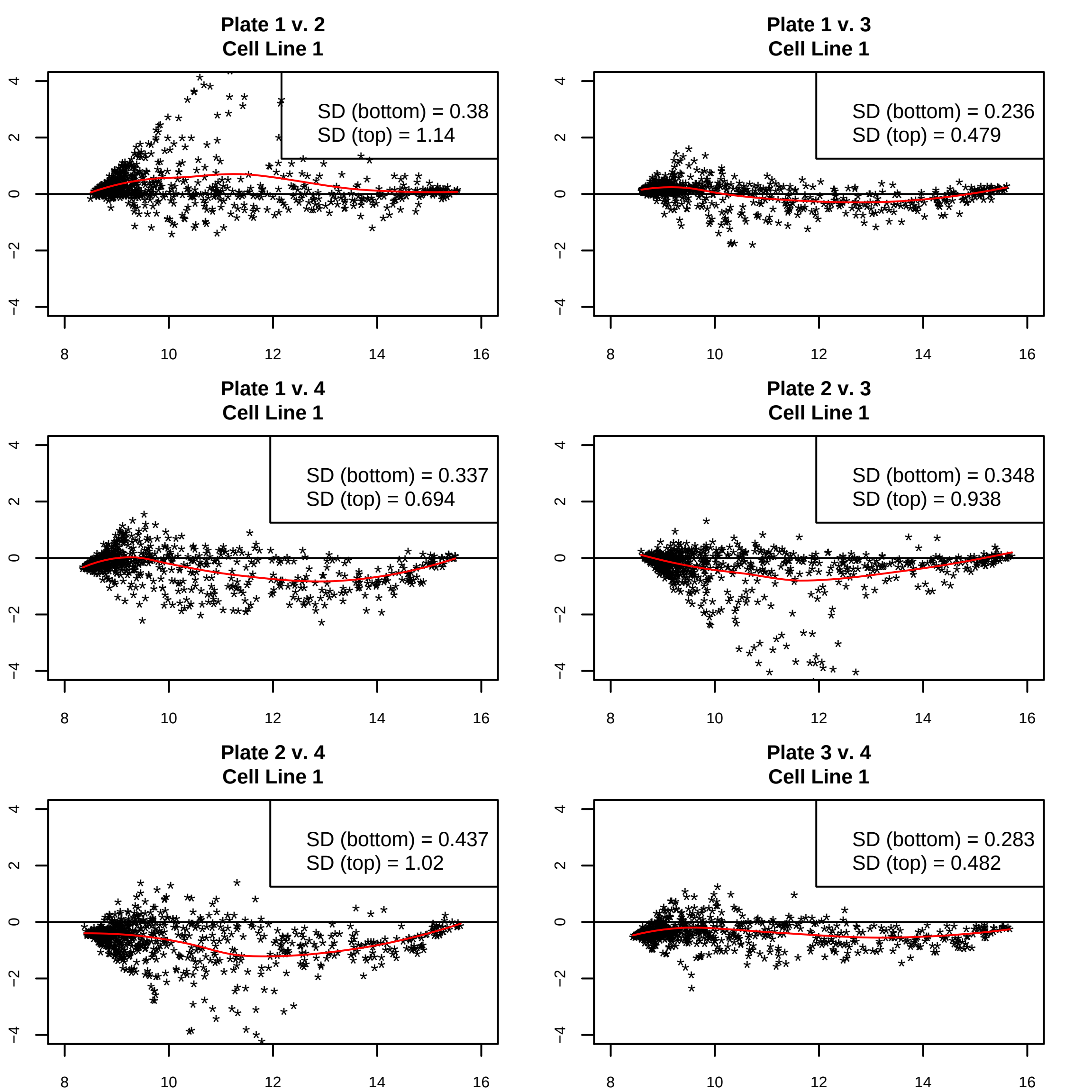

Plate 1 v. 2
Cell Line 1
SD (bottom) = 0.38
SD (top) = 1.14
Plate 1 v. 3
Cell Line 1
SD (bottom) = 0.236
SD (top) = 0.479
Plate 1 v. 4
Cell Line 1
SD (bottom) = 0.337
SD (top) = 0.694
Plate 2 v. 3
Cell Line 1
SD (bottom) = 0.348
SD (top) = 0.938
Plate 2 v. 4
Cell Line 1
SD (bottom) = 0.437
SD (top) = 1.02
Plate 3 v. 4
Cell Line 1
SD (bottom) = 0.283
SD (top) = 0.482

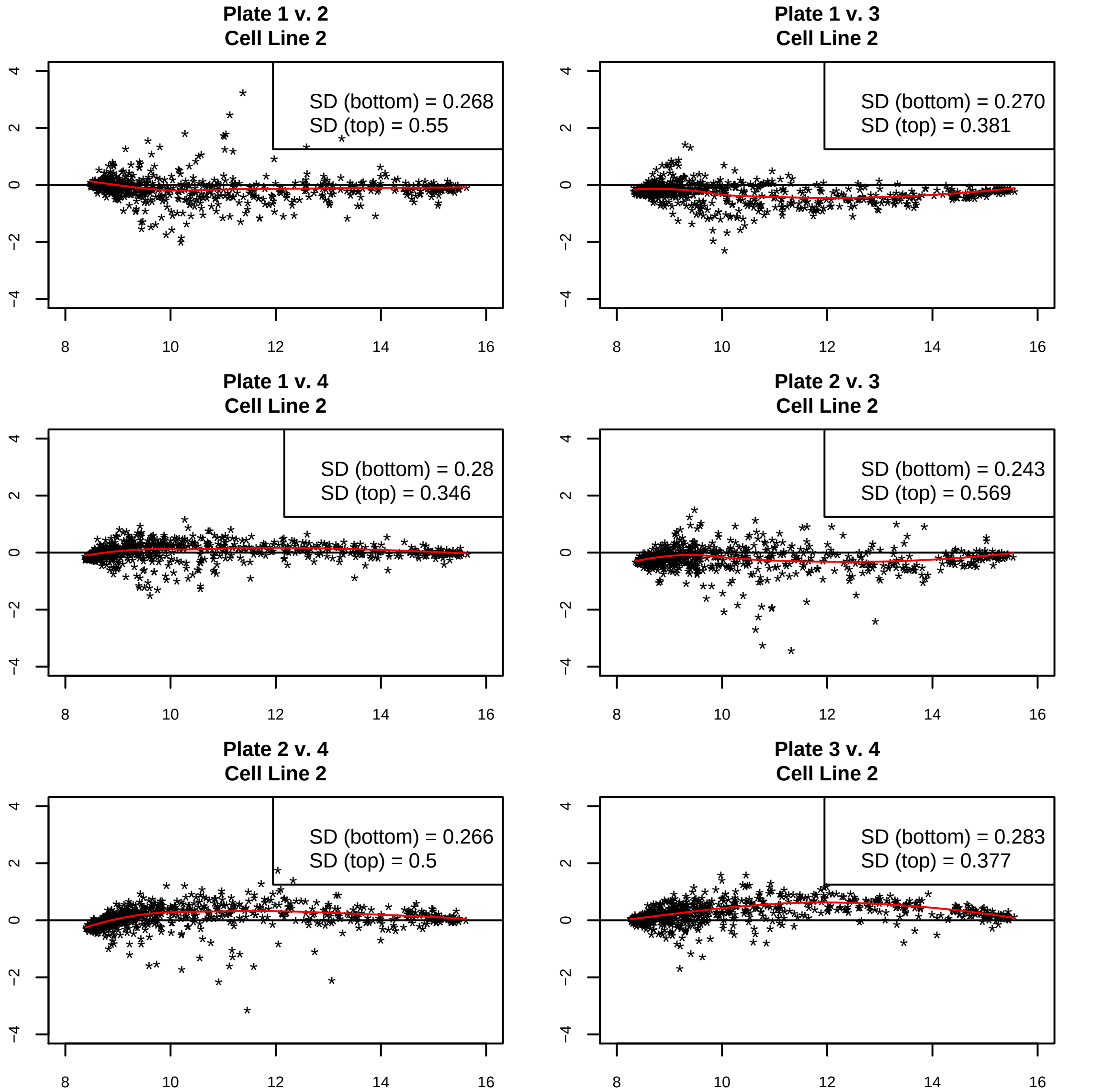
Plate 1 v. 2
Cell Line 2
SD (bottom) = 0.268
SD (top) = 0.55
Plate 1 v. 3
Cell Line 2
SD (bottom) = 0.270
SD (top) = 0.381
Plate 1 v. 4
Cell Line 2
SD (bottom) = 0.28
SD (top) = 0.346
Plate 2 v. 3
Cell Line 2
SD (bottom) = 0.243
SD (top) = 0.569
Plate 2 v. 4
Cell Line 2
SD (bottom) = 0.266
SD (top) = 0.5
Plate 3 v. 4
Cell Line 2
SD (bottom) = 0.283
SD (top) = 0.377
4
2
0
−2
−4
8
10
12
14
16

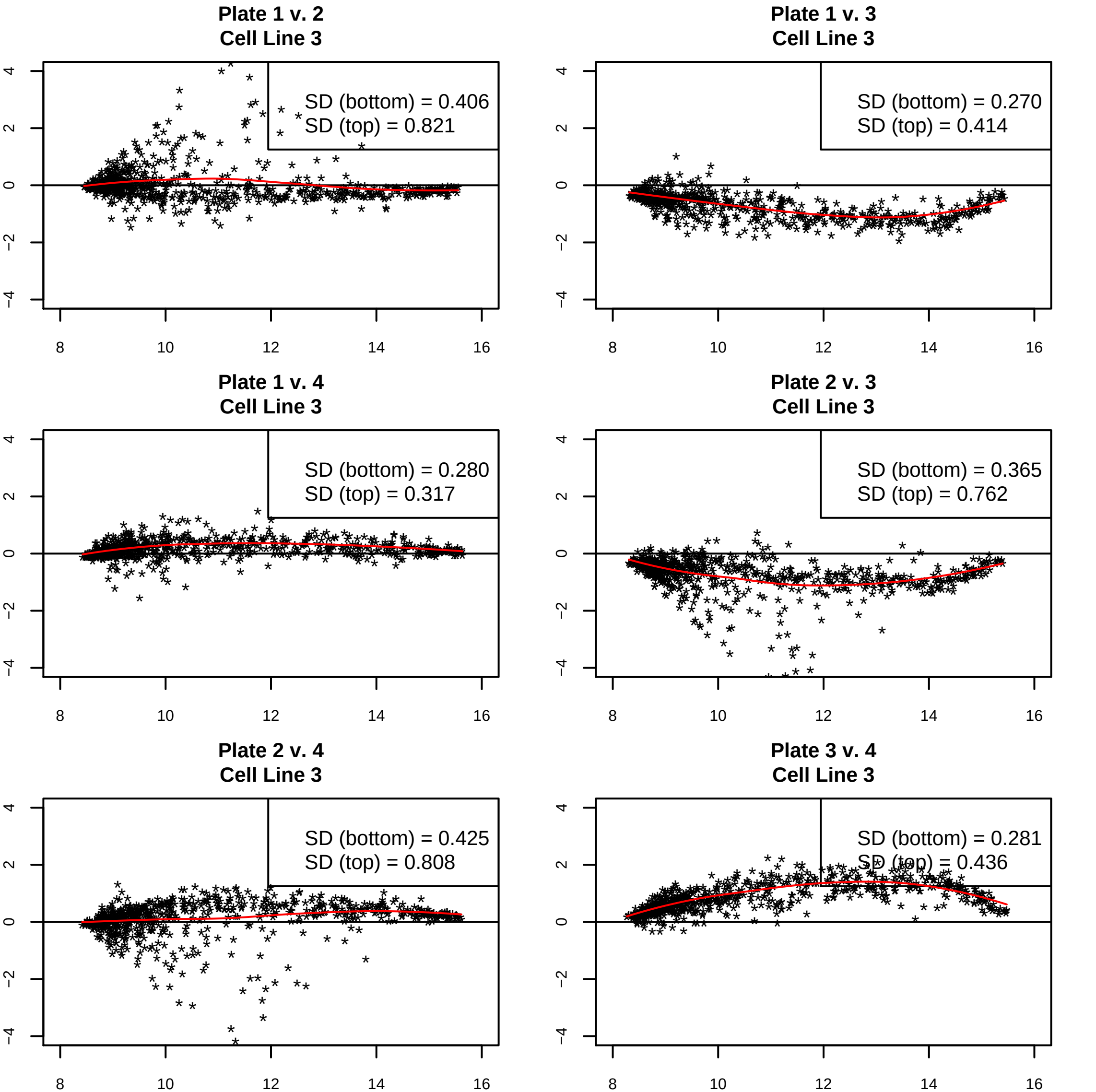
Plate 1 v. 2
Cell Line 3
SD (bottom) = 0.406
SD (top) = 0.821
Plate 1 v. 3
Cell Line 3
SD (bottom) = 0.270
SD (top) = 0.414
Plate 1 v. 4
Cell Line 3
SD (bottom) = 0.280
SD (top) = 0.317
Plate 2 v. 3
Cell Line 3
SD (bottom) = 0.365
SD (top) = 0.762
Plate 2 v. 4
Cell Line 3
SD (bottom) = 0.425
SD (top) = 0.808
Plate 3 v. 4
Cell Line 3
SD (bottom) = 0.281
SD (top) = 0.436
−4
−2
0
2
4
8
10
12
14
16

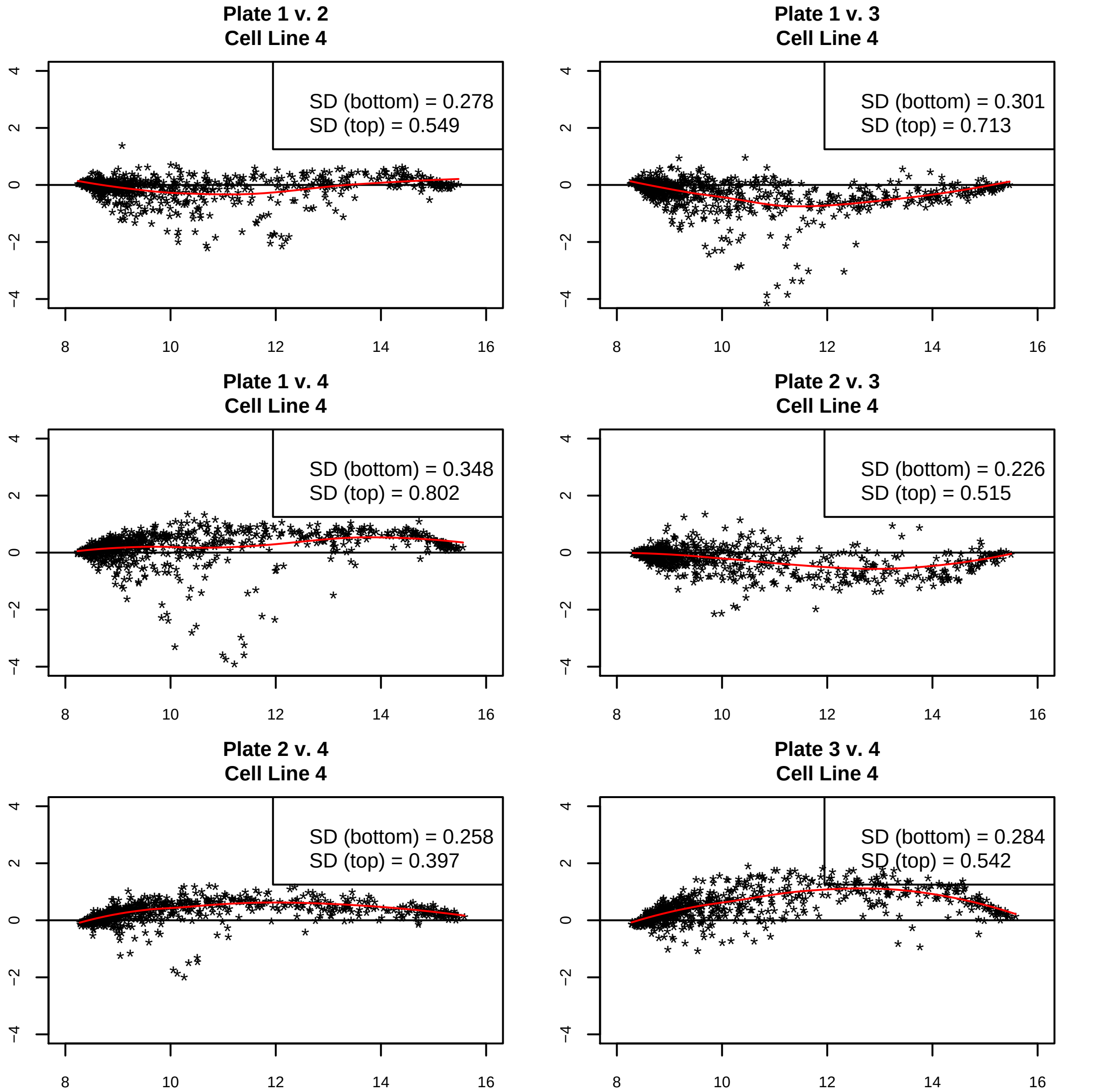

Plate 1 v. 2
Cell Line 4
SD (bottom) = 0.278
SD (top) = 0.549
Plate 1 v. 3
Cell Line 4
SD (bottom) = 0.301
SD (top) = 0.713
Plate 1 v. 4
Cell Line 4
SD (bottom) = 0.348
SD (top) = 0.802
Plate 2 v. 3
Cell Line 4
SD (bottom) = 0.226
SD (top) = 0.515
Plate 2 v. 4
Cell Line 4
SD (bottom) = 0.258
SD (top) = 0.397
Plate 3 v. 4
Cell Line 4
SD (bottom) = 0.284
SD (top) = 0.542
−4
−2
0
2
4
8
10
12
14
16
