# Supplementary material for: Evaluation of a new high-dimensional miRNA profiling platform
Source: BMC Med Genomics. 2009 Aug 27;2:57. doi: 10.1186/1755-8794-2-57 (PMC2744682; doi:10.1186/1755-8794-2-57)
Supplement: Additional file 4 — MVA plots: between plate patient replicates. Pre-normalization MVA plots for 200 ng between SAM patient technical replicates for 200 ng of extraction 1 corresponding to panel D of Figures 3 and 4. Axes are described in the manuscript. [file 1755-8794-2-57-S4.pdf]

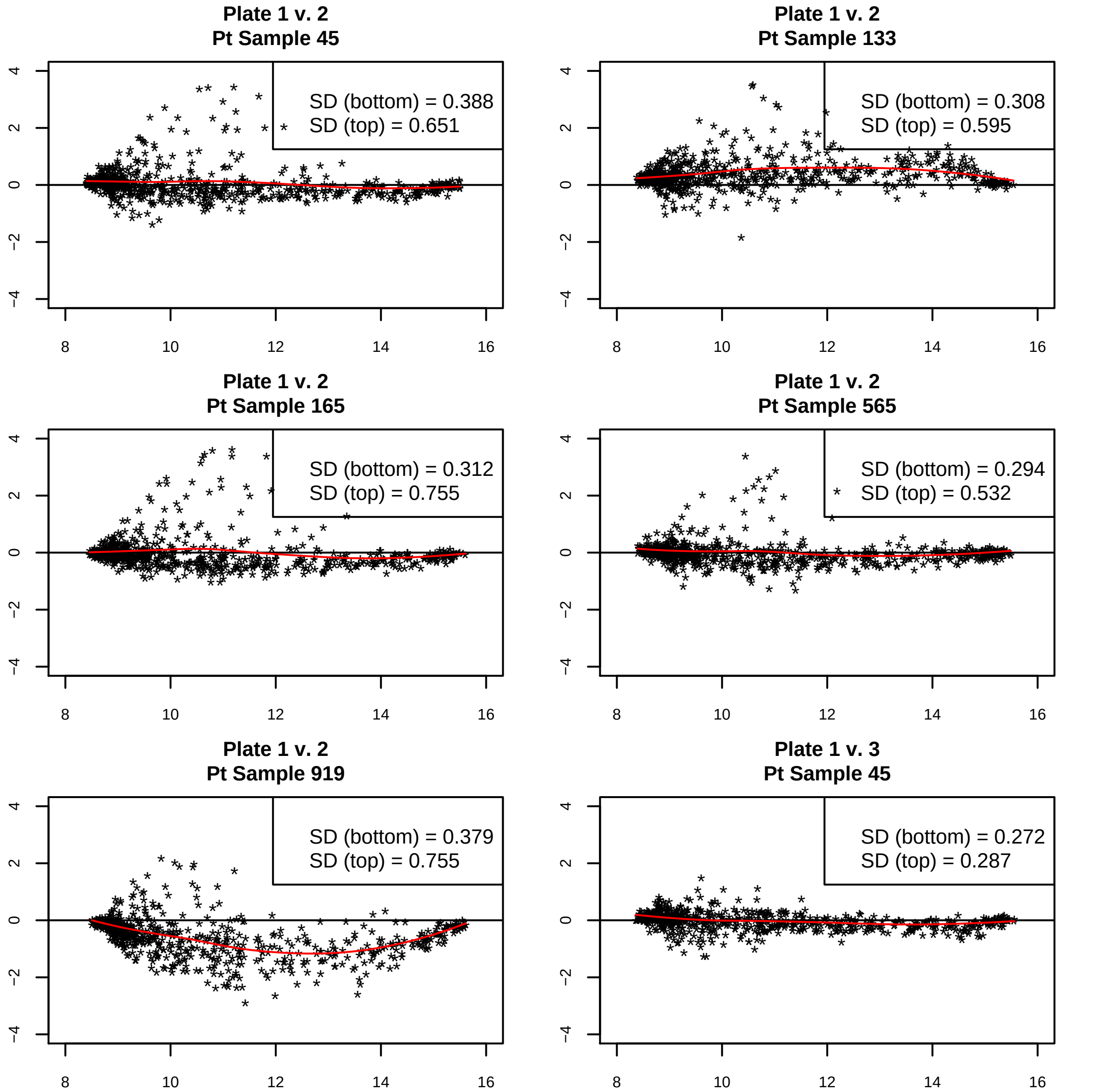

Plate 1 v. 2
Pt Sample 45
SD (bottom) = 0.388
SD (top) = 0.651
Plate 1 v. 2
Pt Sample 133
SD (bottom) = 0.308
SD (top) = 0.595
Plate 1 v. 2
Pt Sample 165
SD (bottom) = 0.312
SD (top) = 0.755
Plate 1 v. 2
Pt Sample 565
SD (bottom) = 0.294
SD (top) = 0.532
Plate 1 v. 2
Pt Sample 919
SD (bottom) = 0.379
SD (top) = 0.755
Plate 1 v. 3
Pt Sample 45
SD (bottom) = 0.272
SD (top) = 0.287
-4
-2
0
2
4
8
10
12
14
16

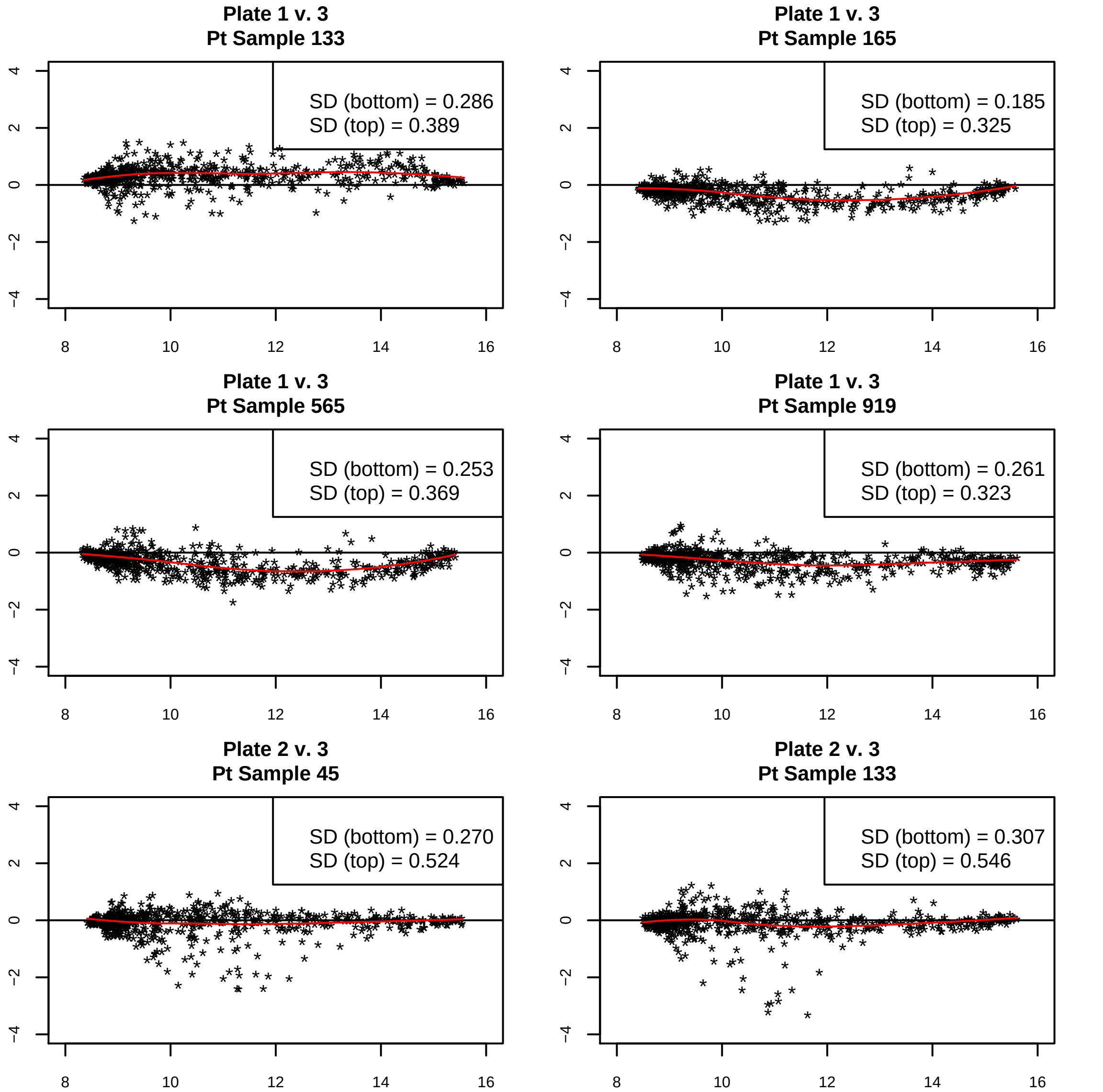
Plate 1 v. 3
Pt Sample 133
SD (bottom) = 0.286
SD (top) = 0.389
Plate 1 v. 3
Pt Sample 165
SD (bottom) = 0.185
SD (top) = 0.325
Plate 1 v. 3
Pt Sample 565
SD (bottom) = 0.253
SD (top) = 0.369
Plate 1 v. 3
Pt Sample 919
SD (bottom) = 0.261
SD (top) = 0.323
Plate 2 v. 3
Pt Sample 45
SD (bottom) = 0.270
SD (top) = 0.524
Plate 2 v. 3
Pt Sample 133
SD (bottom) = 0.307
SD (top) = 0.546
−4 −2 0 2 4
8 10 12 14 16

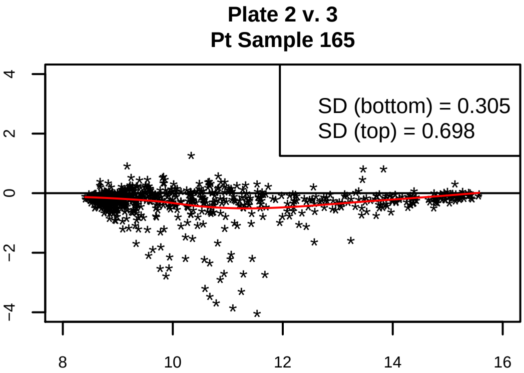
Plate 2 v. 3
Pt Sample 165
SD (bottom) = 0.305
SD (top) = 0.698
4
2
0
−2
−4
8
10
12
14
16

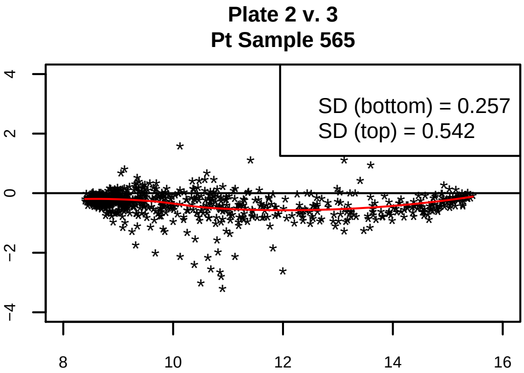
Plate 2 v. 3
Pt Sample 565
SD (bottom) = 0.257
SD (top) = 0.542
4
2
0
−2
−4
8
10
12
14
16

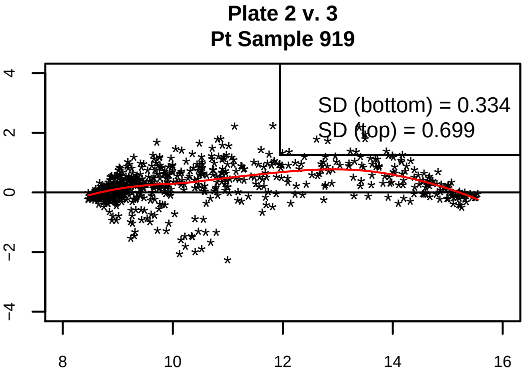
Plate 2 v. 3
Pt Sample 919
SD (bottom) = 0.334
SD (top) = 0.699
4
2
0
−2
−4
8
10
12
14
16
