# Supplementary material for: Evaluation of a new high-dimensional miRNA profiling platform
Source: BMC Med Genomics. 2009 Aug 27;2:57. doi: 10.1186/1755-8794-2-57 (PMC2744682; doi:10.1186/1755-8794-2-57)
Supplement: Additional file 5 — MVA plots: between extractions. Pre-normalization MVA plots for 200 ng between extraction patient technical replicates for 200 ng corresponding to panel E of Figures 3 and 4. Axes are described in the manuscript. [file 1755-8794-2-57-S5.pdf]

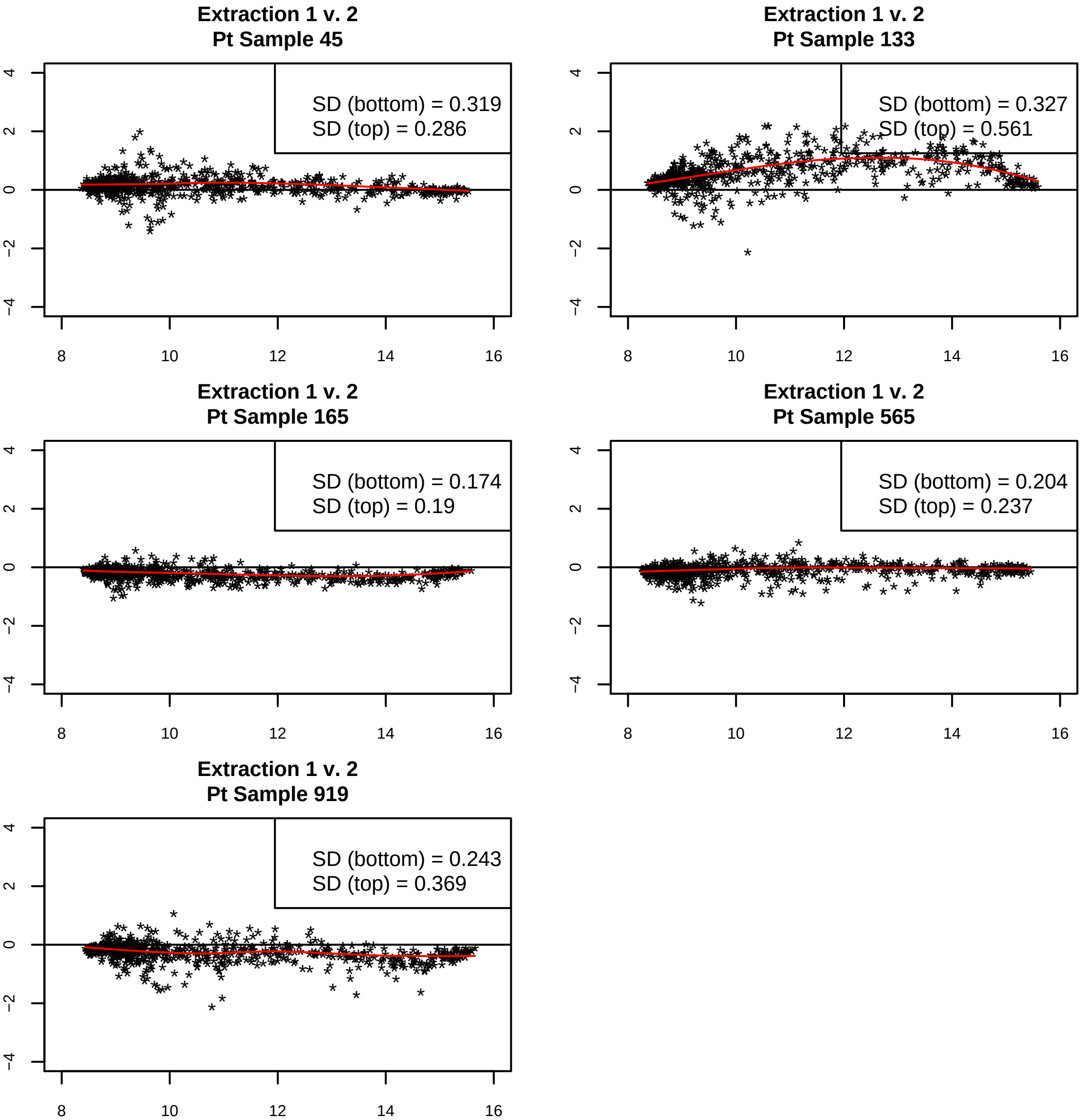
Extraction 1 v. 2
Pt Sample 45
SD (bottom) = 0.319
SD (top) = 0.286
Extraction 1 v. 2
Pt Sample 133
SD (bottom) = 0.327
SD (top) = 0.561
Extraction 1 v. 2
Pt Sample 165
SD (bottom) = 0.174
SD (top) = 0.19
Extraction 1 v. 2
Pt Sample 565
SD (bottom) = 0.204
SD (top) = 0.237
Extraction 1 v. 2
Pt Sample 919
SD (bottom) = 0.243
SD (top) = 0.369
4
2
0
−2
−4
8
10
12
14
16
