# Supplementary material for: Evaluation of a new high-dimensional miRNA profiling platform
Source: BMC Med Genomics. 2009 Aug 27;2:57. doi: 10.1186/1755-8794-2-57 (PMC2744682; doi:10.1186/1755-8794-2-57)
Supplement: Additional file 6 — MVA plots: between dilutions. Pre-normalization MVA plots for patient samples from extraction 1 comparing 25, 100, 400, 800 ng to 200 ng for two patients corresponding to panel F of Figures 3 and 4. Axes are described in the manuscript. [file 1755-8794-2-57-S6.pdf]

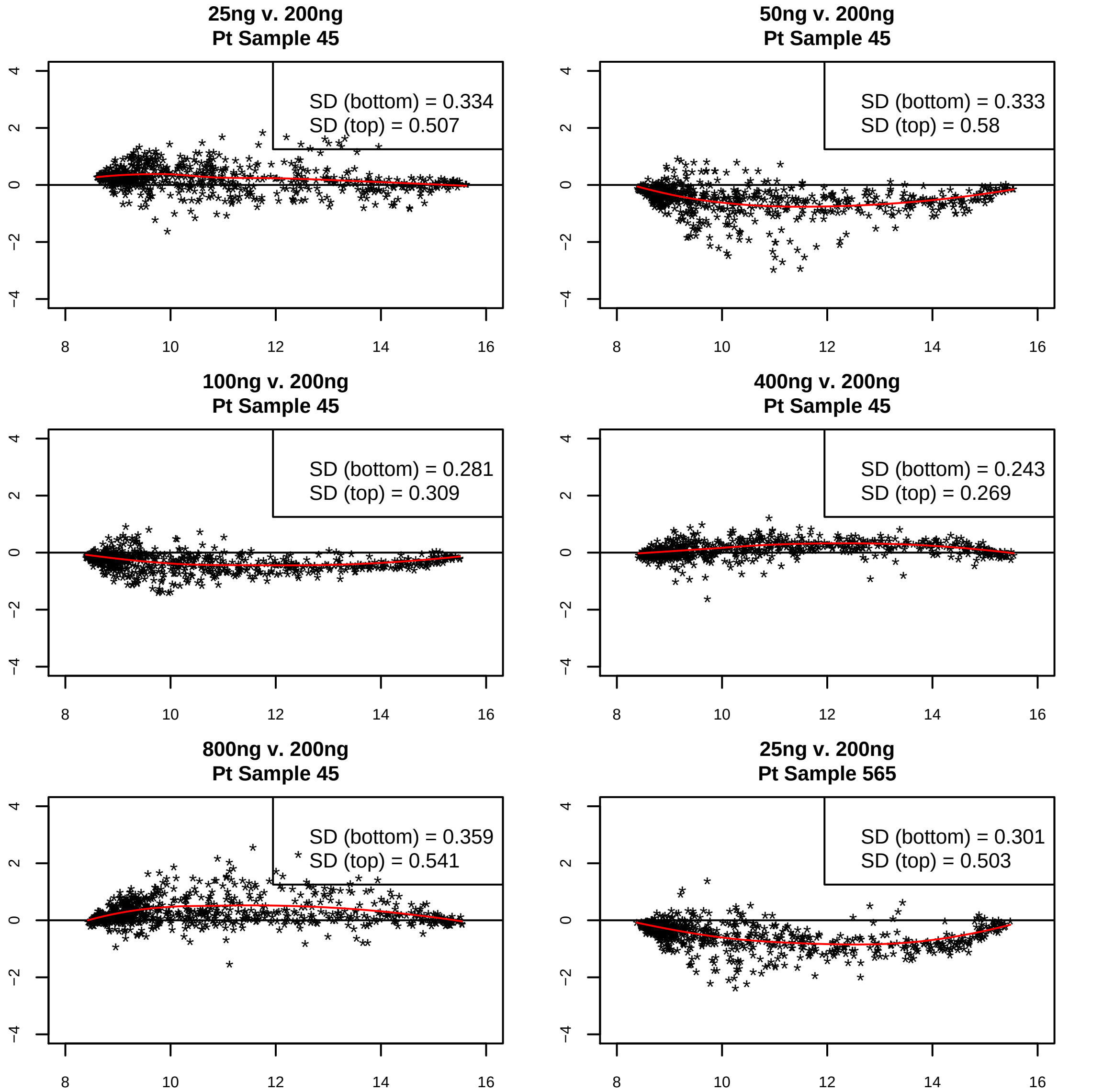

25ng v. 200ng
Pt Sample 45
SD (bottom) = 0.334
SD (top) = 0.507
50ng v. 200ng
Pt Sample 45
SD (bottom) = 0.333
SD (top) = 0.58
100ng v. 200ng
Pt Sample 45
SD (bottom) = 0.281
SD (top) = 0.309
400ng v. 200ng
Pt Sample 45
SD (bottom) = 0.243
SD (top) = 0.269
800ng v. 200ng
Pt Sample 45
SD (bottom) = 0.359
SD (top) = 0.541
25ng v. 200ng
Pt Sample 565
SD (bottom) = 0.301
SD (top) = 0.503
−4 −2 0 2 4
8 10 12 14 16

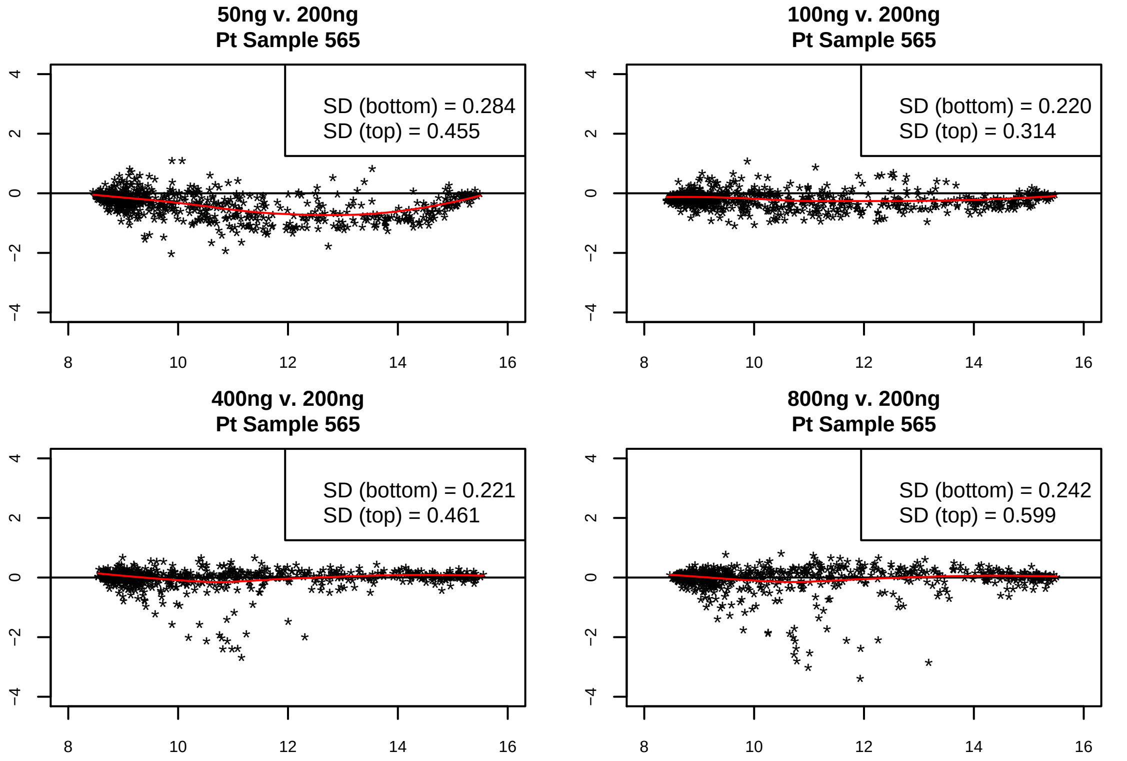
50ng v. 200ng
Pt Sample 565
SD (bottom) = 0.284
SD (top) = 0.455
100ng v. 200ng
Pt Sample 565
SD (bottom) = 0.220
SD (top) = 0.314
400ng v. 200ng
Pt Sample 565
SD (bottom) = 0.221
SD (top) = 0.461
800ng v. 200ng
Pt Sample 565
SD (bottom) = 0.242
SD (top) = 0.599
4
2
0
−2
−4
8
10
12
14
16
