# Supplementary figures and images for: Evaluation of a new high-dimensional miRNA profiling platform
Source: BMC Med Genomics. 2009 Aug 27;2:57. doi: 10.1186/1755-8794-2-57 (PMC2744682; doi:10.1186/1755-8794-2-57)

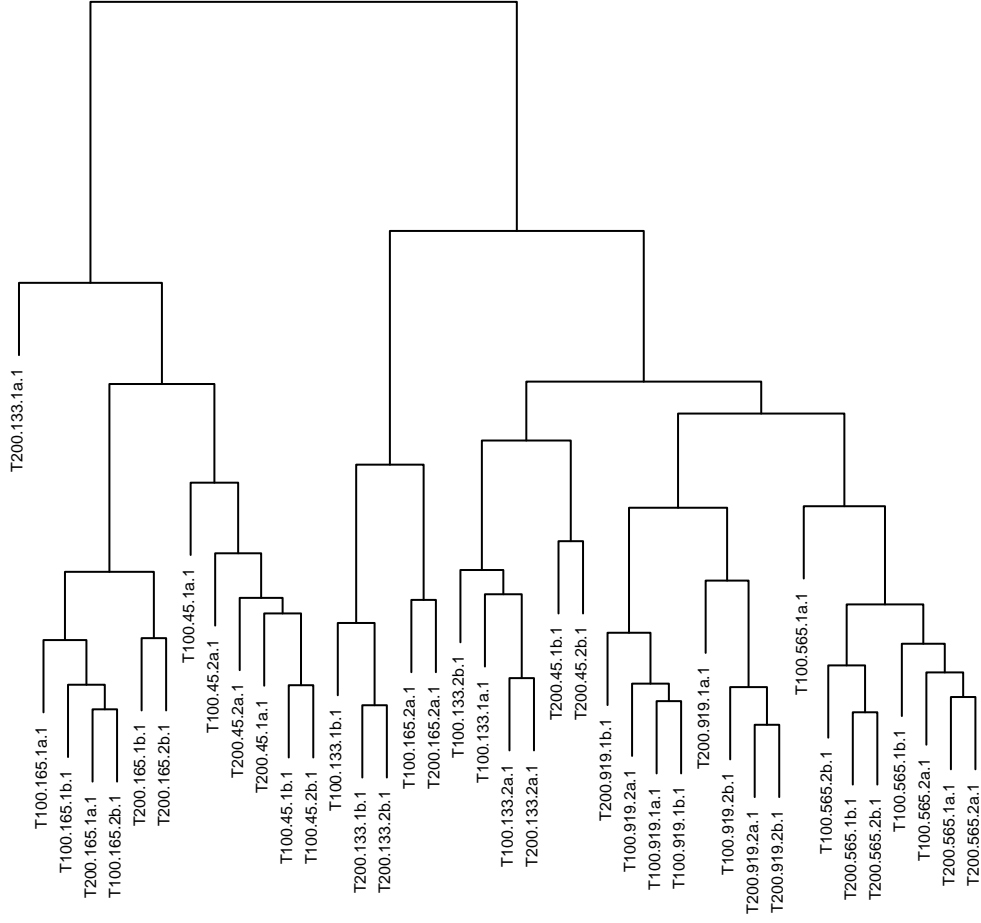

Supplement: Additional file 7 — Pre-normalization clustering dendrogram, plate 1. Pre-normalization dendrogram depicting the results of clustering performed on patient samples on plate 1. Sample IDs are of the form Tdilution.ptID.extraction.replicate. For example, T200.133.1a.1 represents the 200 ng dilution for patient 133 from extraction 1, replicate 1. [file 1755-8794-2-57-S7.pdf]

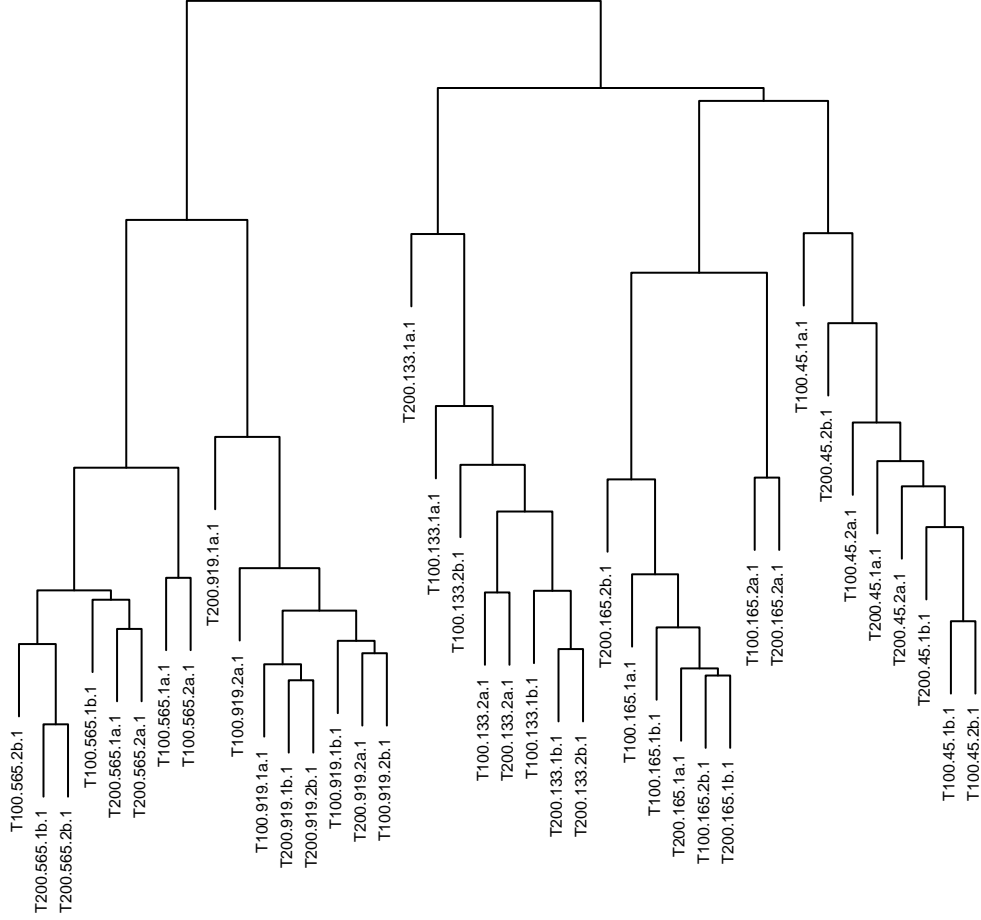

Supplement: Additional file 8 — Post-normalization clustering dendrogram, plate 1. Post-normalization dendrogram depicting the results of clustering performed on patient samples on plate 1. Sample IDs are as described for Additional file 7. [file 1755-8794-2-57-S8.pdf]

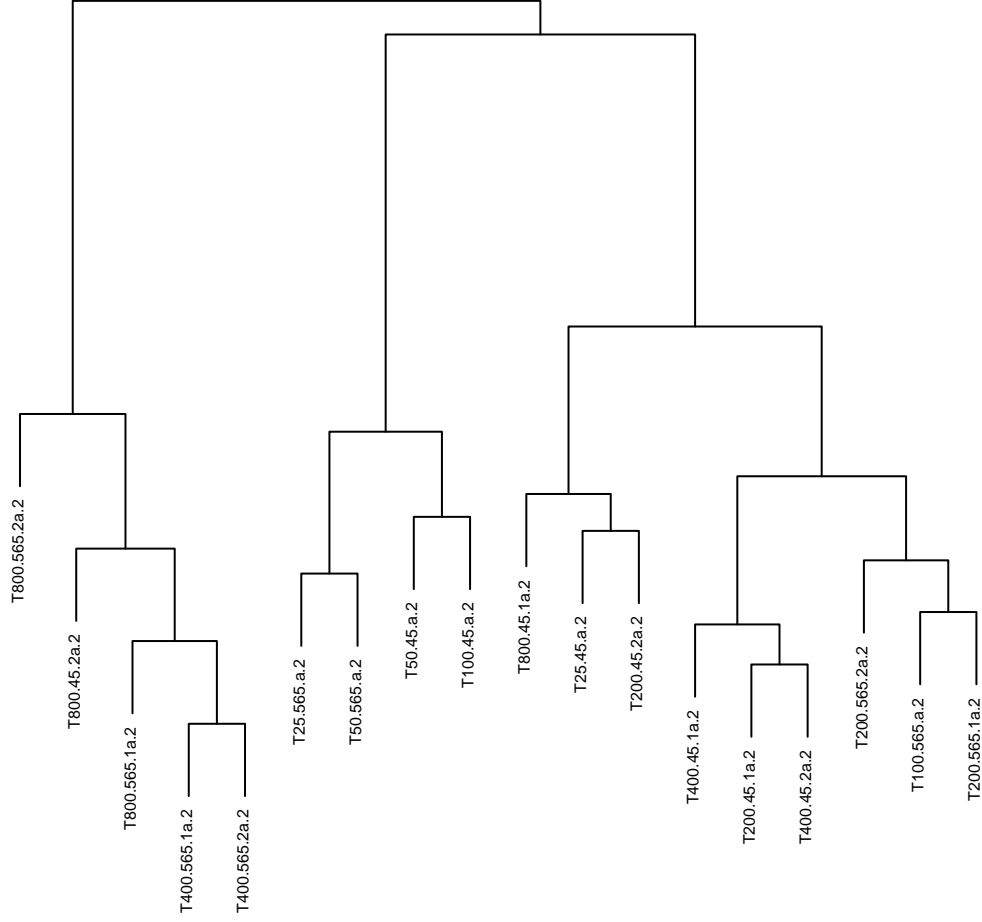

Supplement: Additional file 9 — Pre-normalization clustering dendrogram, plate 2. Pre-normalization dendrogram depicting the results of clustering performed on patient samples on plate 2. Sample IDs are as described for Additional file 7. [file 1755-8794-2-57-S9.pdf]

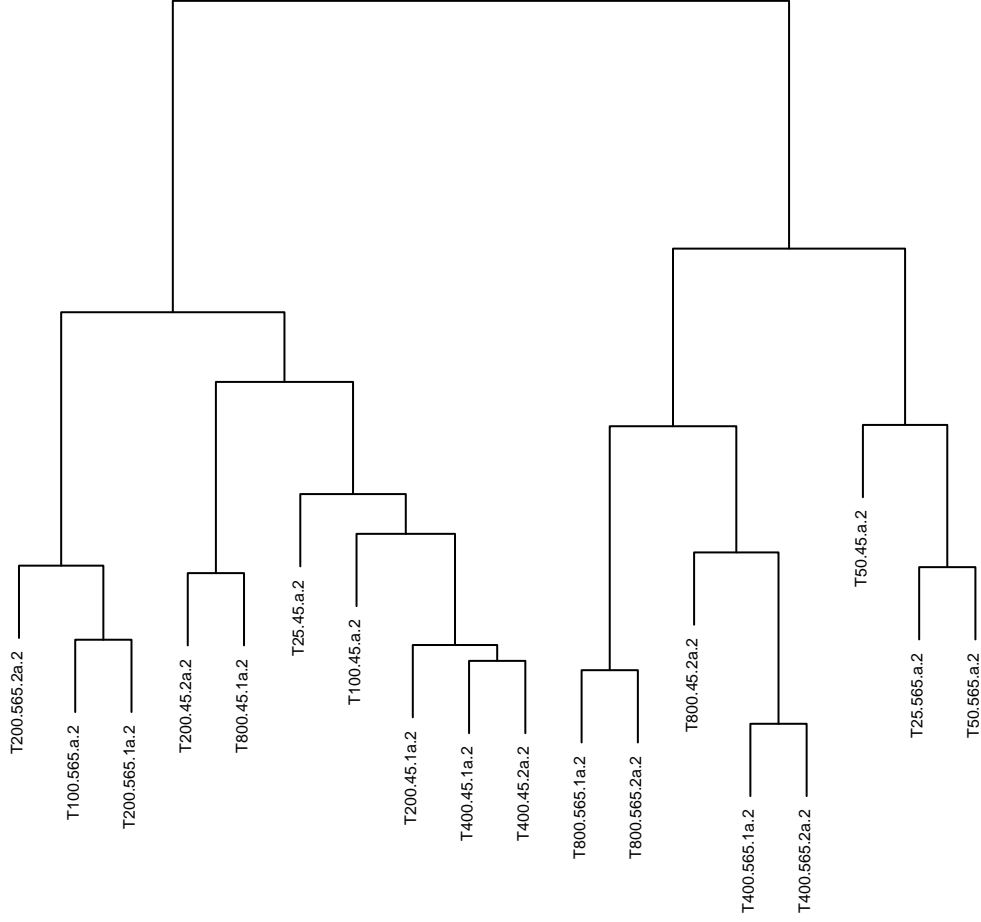

Supplement: Additional file 10 — Post-normalization clustering dendrogram, plate 2. Post-normalization dendrogram depicting the results of clustering performed on patient samples on plate 2. Sample IDs are as described for Additional file 7. [file 1755-8794-2-57-S10.pdf]
