# Supplementary material for: Evaluation of a new high-dimensional miRNA profiling platform
Source: BMC Med Genomics. 2009 Aug 27;2:57. doi: 10.1186/1755-8794-2-57 (PMC2744682; doi:10.1186/1755-8794-2-57)

Patient 45

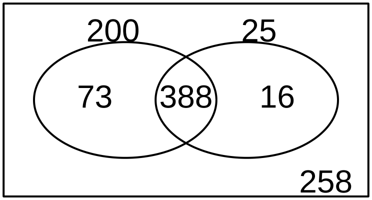

Patient 45

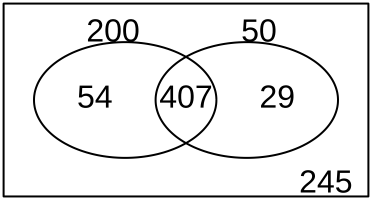

Patient 45

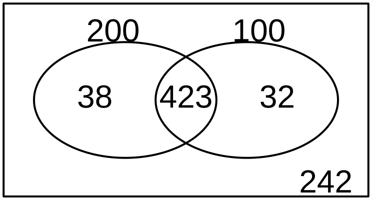

Patient 45

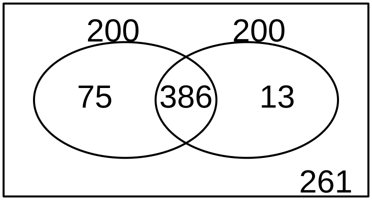

Patient 45

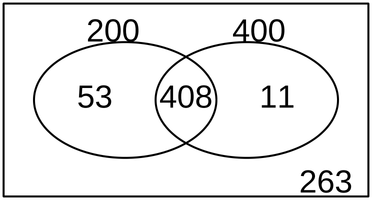

Patient 45

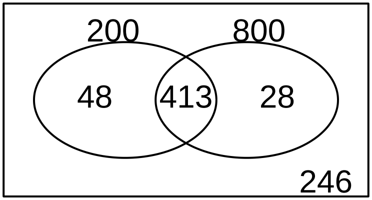

Patient 565

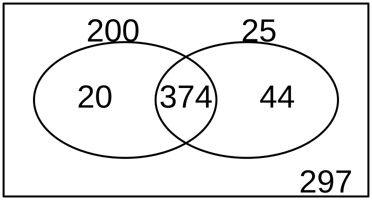

Patient 565

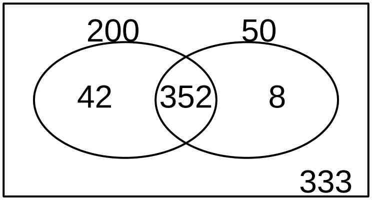

Patient 565

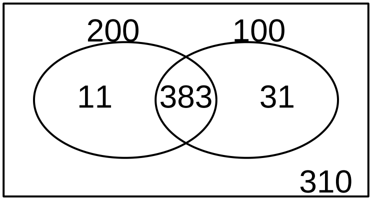

Patient 565

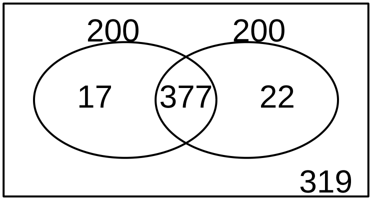

Patient 565

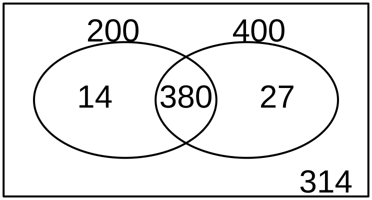

Patient 565

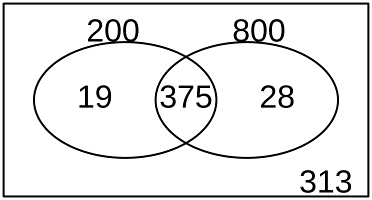

Supplement: Additional file 11 — Venn diagrams of signal detection between replicates. Venn diagrams showing overlap in detection calls between each dilution and the 200 ng replicate 1 for both patients with dilution replicates on SAM 2. Each box represents comparison of one dilution versus the 200 ng replicate 1 sample, where the dilution is labeled on the tops of circles within a box. The numbers inside the circles indicate the number of probes detected in the 200 dilution, both dilutions, or the comparison using the p = 0.01 cut-off to determine detection. The numbers in the bottom right of each box indicate the number of probes not detected in either dilution. For example, for patient 45 comparing 200 ng versus 25 ng, 388 probes were detected in both dilutions, 73 or 16 probes were detected in only the 200 ng or 25 ng dilution, respectively, and 258 were not detected in either dilution. [file 1755-8794-2-57-S11.pdf]
